# Multicenter epidemiological survey of pneumatosis intestinalis in Japan

**DOI:** 10.1186/s12876-022-02343-5

**Published:** 2022-05-31

**Authors:** Naoki Ohmiya, Ichiro Hirata, Hirotsugu Sakamoto, Toshifumi Morishita, Eiko Saito, Katsuyoshi Matsuoka, Tadanobu Nagaya, Shinji Nagata, Miyuki Mukae, Koji Sano, Takayoshi Suzuki, Ken-ichi Tarumi, Seiji Shimizu, Kousaku Kawashima, Toshifumi Hibi, Intractable Diseases, the Health and Labour Sciences Research Group, Naoki Ohmiya, Ichiro Hirata, Hirotsugu Sakamoto, Toshifumi Morishita, Eiko Saito, Katsuyoshi Matsuoka, Tadanobu Nagaya, Shinji Nagata, Miyuki Mukae, Koji Sano, Takayoshi Suzuki, Ken-ichi Tarumi, Seiji Shimizu, Kousaku Kawashima, Toshifumi Hibi, Akimichi Imamura, Yohei Minato, Kazuhiro Matsueda, Go Kuwata, Masahiro Sakaguchi, Daisuke Saito, Sakae Mikami, Mitsuhiro Fujishiro, Shigehiko Fujii, Junji Umeno, Kenji Aoi, Daisuke Nutahara, Fukunori Kinjo, Mikihiro Fujiya, Keita Harada, Mitsunobu Matsushita, Toshimi Chiba, Yutaka Sasaki, Shinji Tanaka, Yoshiaki Aomi, Kunio Kasugai, Shojiro Yamamoto, Nobuaki Yagi, Tomoo Yoshie, Masaki Yoshida, Shin Fukudo, Takanori Yamada, Kensuke Kitsugi, Shigeru Kuriyama, Soichiro Miura, Yoshiya Fujimoto, Yasumasa Niwa, Takashi Nishikawa, Kiyotaka Okawa, Makoto Sanomura, Masanao Nakamura, Tsutomu Mizoshita

**Affiliations:** 1 Departments of Gastroenterology and Advanced Endoscopy, Fujita Health University School of Medicine, 1-98 Kutsukake-cho, Toyoake, Aichi 470-1192 Japan; 2 Department of Medicine, Division of Gastroenterology, Jichi Medical University, Tochigi, Japan; 3 Division of Gastroenterology, Matsuyama Red Cross Hospital, Matsuyama, Japan; 4 Department of Gastroenterology and Hepatology, Tokyo Medical and Dental University, Tokyo, Japan; 5 Division of Gastroenterology and Hepatology, Department of Internal Medicine, Keio University School of Medicine, Tokyo, Japan; 6 Department of Gastroenterology, Shinshu University School of Medicine, Matsumoto, Nagano, Japan; 7 Department of Gastroenterology, Hiroshima City Asa Citizens Hospital, Hiroshima, Japan; 8 Department of Gastroenterology, Kitasato University, School of Medicine, Kanagawa, Japan; 9 Department of Gastroenterology, Osaka City General Hospital, Osaka, Japan; 10 Department of Gastroenterology, Tokai University School of Medicine, Kanagawa, Japan; 11 Division of Gastroenterology, Department of Internal Medicine, Kawasaki Medical School, Kurashiki, Okayama Japan; 12 Departments of Gastroenterology and Hepatology, Osaka General Hospital of West Japan Railway Company, Osaka, Japan; 13 Department of Gastroenterology, Shimane University School of Medicine, Izumo, Shimane, Japan; 14 Center for Advanced IBD Research and Treatment, Kitasato Institute Hospital, Kitasato University, Tokyo, Japan

**Keywords:** Pneumatosis intestinalis, Small bowel, Complications, Poor prognosis

## Abstract

**Background:**

Pneumatosis intestinalis (PI) is a rare condition characterized by gas collection in the intestinal wall. We aimed to determine the etiology and affected segments associated with complications, treatment, and outcome.

**Methods:**

We conducted a multicenter epidemiological survey using a standardized data collection sheet in Japan. Complicating PI was defined as strangulation or bowel necrosis, bowel obstruction, adynamic ileus, sepsis, shock, and massive gastrointestinal bleeding requiring blood transfusion.

**Results:**

We enrolled 167 patients from 48 facilities. Multivariate analysis revealed that older age (adjusted OR, 1.05 and 95% confidence intervals [CI], 1.02–1.09, *P* = 0.0053) and chronic kidney disease (adjusted OR, 13.19 and 95% CI 1.04–167.62, *P* = 0.0468) were independent predictors of the small-bowel-involved type. Complicating PI was associated with the small-bowel-involved combined type (adjusted OR, 27.02 and 95% CI 4.80–152.01, *P* = 0.0002), the small-bowel-only type (adjusted OR, 3.94 and 95% CI 1.02–15.27, *P* = 0.0472), and symptomatic PI (adjusted OR, 16.24 and 95% CI 1.82–145.24, *P* = 0.0126). Oxygen therapy was performed in patients with a past history of bowel obstruction (adjusted OR, 13.77 and 95% CI 1.31–144.56, *P* = 0.0288) and surgery was performed in patients with complicating PI (adjusted OR, 8.93 and 95% CI 1.10–72.78, *P* = 0.0408). Antihypertensives (adjusted OR, 12.28 and 95% CI 1.07–140.79, *P* = 0.0439) and complicating PI (adjusted OR, 11.77 and 95% CI 1.053–131.526; *P* = 0.0453) were associated with exacerbation of PI. The complicating PI was the only indicator of death (adjusted OR, 14.40 and 95% CI 1.09–189.48, *P* = 0.0425).

**Discussion:**

Small-bowel-involved type and symptomatic PI were associated with complications which were indicators of poor prognosis.

**Supplementary Information:**

The online version contains supplementary material available at 10.1186/s12876-022-02343-5.

## Introduction

Pneumatosis intestinalis (PI) is a rare condition characterized by the collection of gas, which can have a hydrogen content up to 50%, in the intestinal wall [1, 2]. PI has been classified pathogenically into four categories: bowel necrosis, mucosal disruption, increased mucosal permeability, and pulmonary disease [2]. The relevant literature from Japan has described 12 patients with PI due to chronic occupational exposure to trichloroethylene [3], 14 following treatment of diabetes mellitus with α-glucosidase inhibitors [4], and 39 with systemic sclerosis [5]. An American multicenter retrospective study including 500 PI patients reported that 40% had transmural ischemia or withdrawal of clinical care and subsequent mortality, while 60% had benign diseases that could be observed without any aggressive intervention [6]. In Asia, a Korean retrospective study including 123 PI patients demonstrated that the most common cause was mesenteric vascular ischemia (35.0%), followed by bowel obstruction (13.8%), chemotherapy (10.6%), adynamic ileus (7.3%), post-anastomosis (3.3%), chronic obstructive pulmonary disease (2.4%), and nonspecific enteritis (2.4%) [7].

PI is distributed throughout the digestive tract, especially in the small and large intestine, and PI is occasionally accompanied by pneumoperitoneum and portal venous gas. The clinical significance of PI varies from an incidental finding that resolves spontaneously to death. A multicenter prospective epidemiologic study of the American Association for the Surgery of Trauma including 127 patients with PI recommended that surgical exploration be strongly considered for patients presenting with a blood lactate value greater than 2.0 mmol/L (18 mg/dL) and/or peritonitis; furthermore, the authors of the report suggested strong suspicion for necrosis in those patients and small bowel involvement, ascites, adynamic ileus, anemia, and a high international normalized ratio [8]. The large intestine is affected most commonly, while the small intestine is associated with this complicating pathologic PI [6], the mechanism of which is not fully elucidated. In addition, a large-scale multicenter study of PI has not been conducted in Japan to date. Therefore, we conducted a multicenter epidemiological survey including 167 patients with PI in Japan and determined the etiology and affected segments associated with complications, treatment, and outcome.

## Patients and methods

### Study design

This was a retrospective multicenter epidemiological study using a standardized data collection sheet. The diagnosis of PI was made with the identification of characteristic features or gas in the bowel wall by endoscopy, endoscopic ultrasonography, CT, plain abdominal roentgenogram, barium enema roentgenogram, or laparotomy. The aim of this study was to define the clinical features leading to complications and poor prognosis. Complicating PI was defined as having strangulation or bowel necrosis, bowel obstruction, adynamic ileus without mechanical obstruction, sepsis, shock, and massive gastrointestinal bleeding requiring blood transfusion. Benign PI was defined as not having those complications. This study was reviewed and approved by the institutional review board and ethics committee of Fujita Health University Hospital. Informed consent was obtained in the form of opt-out on the web-site. Those who rejected it were excluded. All authors had access to the study data and reviewed and approved the final manuscript.

#### Survey

The data collection sheet comprised questions on demographics, medical history, medications used, examinations, symptoms, complications, treatment, and outcome, as listed in Additional file 1: Supplementary Table 1. We sent this data sheet to 150 medical facilities in Japan in April 2013 and collected it by August 2013. When some items of the data sheet were unanswered, the numbers of patients subjected to analyses varied among the items.

#### Statistical analysis

The numbers in the text are expressed as the median (range), and comparisons were analyzed using the Mann–Whitney U test. The proportions of patients among categorical items were compared by Fisher’s exact probability test or the χ^2^ test. Multivariate analysis was performed using logistic regression analysis for categorical items with *P*-values less than 0.10 in the univariate analysis. Categorical items were excluded for multivariate analysis unless the number of patients in either of groups exceeded 2. To elucidate the relationship between the segments involved and clinical characteristics, the segments were classified into two types: large bowel-only type and small bowel-involved type. The small-bowel-involved type was classified into the small-bowel-involved combined type with affected segments combining the small bowel and the other gastrointestinal tract, and the small-bowel-only type. Crude odds ratios (ORs) and adjusted ORs with 95% confidence intervals (95% CIs) were computed. Differences were considered significant with *P*-values less than 0.05.

## Results

### Clinical characteristics of pneumatosis intestinalis

Standardized data collection sheets were returned from 48 facilities (32%) including 16 secondary and 32 tertiary health care hospitals, and 167 cases were enrolled. The patients’ demographics, comorbidities, past medical histories, and medications used are shown in Table 1. Symptoms, complications, segments involved, diagnostic examinations, treatment, outcome of PI, and prognosis are shown in Table 2.

**Table 1:** Clinical characteristics of patients with pneumatosis intestinalis

Characteristics	n	(%)
No. patients	167	
Men/women	87/80	
Median age of onset (years)	65 (range 9–91)	
Exposure to organic solvents	2	(1)
Comobidities and/or past medical history	141	(84)
Gastroduodenal diseases	45	(27)
Inflammatory bowel disease	16	(10)
Ulcerative colitis	13	(8)
Crohn's disease	2	(1)
Behcet's disease	1	(1)
Carcinoma	13	(8)
Esophegeal carcinoma	1	(1)
Gastric carcinoma	3	(2)
Colorectal carcinoma	9	(5)
Colorectal polyp	6	(4)
Bowel obstruction	3	(2)
Others	5	(3)
Diabetes mellitus	31	(19)
Chronic lung disease	28	(17)
Autoimmune disease	30	(18)
Hypertension	13	(8)
Heart disease	11	(7)
Hepatobiliarypancreatic disease	10	(6)
Kideny disease	4	(2)
Others	41	(25)
Medications used	137	(82)
Corticosteroid	45	(27)
Antidiabetics	29	(17)
α-glucosidase inhibitors	23	(14)
Others	14	(8)
Gastric acid secretion inhibitors	20	(12)
Proton pump inhibitors	15	(9)
Histamine-2 receptor antagonists	6	(4)
Antihypertensives	19	(11)
Calcium antagonist	10	(6)
β-blocker	8	(5)
Angiotensin II receptor blocker	9	(5)
Angiotensin converting enzyme inhibitor	1	(1)
α-blocker	1	(1)
Immunosuppressants	16	(10)
5-aminosalicylates or salicylazosulfapyridine	15	(9)
Statins/ezetimib/fibrates	14	(8)
Antithrombotics	12	(7)
Anticoagulants	4	(2)
Antiplatelets	9	(5)
Laxatives*	10	(6)
Anti-cancer agents	9	(5)
Herbal medicine	7	(4)
Allopurinol/benzbromaron	3	(2)
Others	44	(26)

*Magnesium oxide (n = 7), sennoside (n = 4), and sodium picosulfate (n = 4)

**Table 2:** Pneumatosis intestinalis (n = 167)

Characteristics	n	(%)
Symptoms		
Asymtomatic	70	(41.9)
Symptomatic	97	(58.1)
Complications		
Abscense	141	(84.4)
Presence	26	(15.6)
Strangulation or bowel necrosis	10	(6.0)
Bowel obstruction	4	(2.4)
Massive bleeding	4	(2.4)
Adynamic ileus	4	(2.4)
Bowel perforation	3	(1.8)
Sepsis	1	(0.6)
Segments involved		
Large bowel only	119	(71.3)
Right-sided colon only	84	(50.3)
Left-sided colon only	25	(15.0)
Right- and left-sided colon	6	(3.6)
Throughout the large bowel	2	(1.2)
Rectum only	1	(0.6)
Left-sided colon and rectum	1	(0.6)
Small bowel only	33	(19.8)
Ileum only	14	(8.4)
Jejunum only	11	(6.6)
Ileum and jejunum	8	(4.8)
Combined	13	(7.8)
Ileum and right-sided colon	3	(1.8)
Ileum, right- and left-sided colon	3	(1.8)
Jejunum, ileum, right- and left-sided colon	3	(1.8)
Esophagus, stomach, small bowel	2	(1.2)
Jejunum and right-sided colon	1	(0.6)
Esophagus, stomach, small bowel, and colon	1	(0.6)
Diagnostic examination		
Endoscopy	90	(53.9)
Computed tomography	85	(50.9)
Plain abdominal roentgenogram	27	(16.2)
Barium enema roentgenogram	19	(11.4)
Endoscopic ultrasonography	6	(3.6)
Laparotomy	1	(0.6)
Treatment		
Conservative treatment	117	(70.1)
Oxygen therapy	35	(21.0)
Hyperbaric	16	(9.6)
Conventional	19	(11.4)
Endoscopic therapy	3	(1.8)
Puncture with needle	2	(1.2)
Deaeration	1	(0.6)
Surgery	12	(7.2)
Outcome of pneumatosis intestinalis		
Improvement	119	(71.3)
Disapperance	80	(47.9)
Reduction	36	(21.6)
Recurrence	1	(0.6)
Unknown	2	(1.2)
No change	40	(24.0)
Exacerbation	8	(4.8)
Prognosis		
Survival	159	(95.2)
Death	8	(4.8)
Strangulation	4	(2.4)
Sepsis	2	(1.2)
Bleeding	1	(0.6)
Data unavailable	1	(0.6)

### Segment of bowel involved

A comparison of clinical characteristics between the large-bowel-only type and the small-bowel-involved type is shown in Additional file 2: Supplementary Table 2. Although univariate analysis demonstrated that older age, non-use of α-glucosidase inhibitors, 5-aminosalicylates, salicylazosulfapyridine, or statins/ezetimib/fibrates, negativity for ulcerative colitis, positivity for autoimmune disease, chronic kidney disease, and cancer other than that of the digestive or hematologic system were associated with the small-bowel-involved type, multivariate analysis revealed that older age (adjusted OR, 1.05 and 95% confidence intervals [CI], 1.02–1.09, *P* = 0.0053) and chronic kidney disease (adjusted OR, 13.19 and 95% CI 1.04–167.62, *P* = 0.0468) were the only independent predictors (Table 3).

**Table 3:** Multivariate analysis of segments, complications, treatment, and outcomes of pneumatosis intestinalis

Characteristics	Adjusted OR	95% CI	*P*
Segment of bowel involved			
Small-bowel-involved type			
Age	**1.05**	**(1.02–1.09)**	**0.0053**
Chronic kideny disease	**13.19**	**(1.04–167.62)**	**0.0468**
a-glucosidase inhibitors	0.27	(0.07–1.01)	0.0519
Complicating pneumatosis intestinalis			
Small-bowel-involved combined type	**27.02**	**(4.80–152.01)**	**0.0002**
Symptomatic	**16.24**	**(1.82–145.24)**	**0.0126**
Small-bowel-only type	**3.94**	**(1.02–15.27)**	**0.0472**
Treatment			
Oxygen therapy			
Past history of bowel obstruction	**13.77**	**(1.31–144.55)**	**0.0288**
Proton pump inhibitors	3.14	(0.83–11.96)	0.0928
Surgery			
Complicating pneumatosis intestinalis	**8.93**	**(1.10–72.78)**	**0.0408**
Small bowel only segment	6.21	(0.74–52.18)	0.9260
Outcomes of pneumatosis intestinalis			
Exacerbation			
Antihypertensives	**12.28**	**(1.07–140.79)**	**0.0439**
Complicating pneumatosis intestinalis	**11.77**	**(1.053–131.526)**	**0.0453**
Prognosis of patients with pneumatosis intestinalis (death)			
Complicating pneumatosis intestinalis	**14.40**	**(1.09–189.48)**	**0.0425**

Bold indicate *P*-values less than 0.05

### Complicating pneumatosis intestinalis

A comparison of clinical characteristics between benign and complicating PI is shown in Additional file 3: Supplementary Table 3. Symptoms of complicating PI included abdominal pain/distention (n = 25, 96%) and bloody stool (n = 4, 15%) except one asymptomatic patient with hydrocephalus after subarachnoid hemorrhage, while those of benign PI included abdominal pain/distention (n = 49, 35%), diarrhea (n = 17, 12%), constipation (n = 10, 7%), and bloody stool (n = 8, 6%). The multivariate analysis revealed that the small-bowel-involved combined type (adjusted OR, 27.02 and 95% CI 4.80–152.01, *P* = 0.0002), symptomatic PI (adjusted OR, 16.24 and 95% CI 1.82–145.24, *P* = 0.0126), and the small-bowel-only type (adjusted OR, 3.94 and 95% CI 1.02–15.27, *P* = 0.0472) were the only independent predictors (Table 3).

### Treatment of pneumatosis intestinalis

The comparison of clinical characteristics in terms of medical treatment, oxygen therapy, endoscopic therapy, and surgery is shown in Additional file 4: Supplementary Table 4. The rates of improvement, exacerbation, recurrence, and death stratified by conservative, oxygen, and surgical treatment were 67.5%, 4.3%, 0.0%, and 5.1%; 80.0%, 2.9%, 2.9%, and 2.9%; and 83.3%, 8.3%, 0.0%, and 8.3%, respectively. Univariate analysis demonstrated the following: non-use of 5-aminosalicylates/salicylazosulfapyridine, a past history of bowel obstruction, autoimmune disease, affected segments other than the large bowel associated with oxygen treatment, use of anticancer agents, the small-bowel-only type and complicating PI associated with surgery. Multivariate analysis demonstrated that oxygen therapy was performed in patients with a past history of bowel obstruction (adjusted OR, 13.77 and 95% CI 1.31–144.56, *P* = 0.0288); and surgery was performed in patients with complicating PI (adjusted OR, 8.93 and 95% CI 1.10–72.78, P = 0.0408), as shown in Table 3.

### Outcome of pneumatosis intestinalis

The comparison of the clinical characteristics among the outcomes of PI, namely, improvement, no change, and exacerbation, is shown in Additional file 5: Supplementary  Table 5. Univariate analysis demonstrated that non-use of α-glucosidase inhibitors and use of 5-aminosalicylates/salicylazosulfapyridine associated with no change, use of antihypertensives, chronic kidney disease, and involved segments other than the large bowel were associated with exacerbation. Multivariate analysis demonstrated that the use of antihypertensives (adjusted OR, 12.28 and 95% CI 1.07–140.79, *P* = 0.0439) and complicating PI (adjusted OR, 11.77 and 95% CI 1.053–131.526; *P* = 0.0453) were associated with exacerbation of PI, as shown in Table 3.

### Death associated with pneumatosis intestinalis

The comparison of clinical characteristics in terms of survival and death associated with PI is shown in Additional file 6: Supplementary Table 6. Although the univariate analysis demonstrated that the use of α-blocker, laxatives, cirrhosis, and chronic kidney disease, the small-bowel-involved type, complicating PI were associated with death, the multivariate analysis revealed that the complicating PI was the only indicator of death (adjusted OR, 14.40 and 95% CI 1.09–189.48, *P* = 0.0425), as shown in Table 3. Of the eight patients, 6 died from complicating PI (4 with bowel infarction, 1 with septic shock, and 1 with massive gastrointestinal bleeding), and 2 died from severe comorbidities such as liver cirrhosis with chronic kidney disease and acute leukemia followed by graft-versus-host disease.

## Discussion

The present multicenter epidemiologic study demonstrated that complicating PI, such as strangulation or bowel necrosis, bowel obstruction, adynamic ileus, sepsis, shock, and massive gastrointestinal bleeding, was significantly associated with the small-bowel-involved combined type, the small-bowel-only type, and symptomatic PI. These results support the findings of another prospective multicenter study including 127 PI patients sponsored by the Association for Surgery of Trauma: the small bowel location of PI, peritonitis, and abnormal laboratory values such as an elevated international normalized ratio, decreased hemoglobin, and lactate values greater than 2.0 mmol/L were predictive of pathologic PI defined as the presence of transmural ischemia during surgical exploration or autopsy [8]. Similarly, the largest-scale retrospective multicenter epidemiologic study of the Eastern Association for the Surgery of Trauma including 500 patients demonstrated that the large bowel disease was the most common site for PI, but the jejunal and ileal pneumatosis locations were most commonly associated with pathologic PI [6]. A retrospective single-center study including 70 patients with PI or portal vein gas-clarified acute mesenteric ischemia was associated with small bowel PI, abdominal pain, elevated lactate, and the calculated vascular disease score [9]. These studies are in line with the present finding, but in a retrospective single-center study including 97 patients with PI (46% colon, 27% stomach, 5% stomach, and 7% both small and large bowel), Morris et al. reported that the location of pneumatosis alone was not predictive of outcome or intervention [10]. The present study has the inherent limitation of its lack of data on blood and physical findings, but a comprehensive diagnosis that includes a physical examination with parameters such as vital and peritoneal signs, laboratory tests, and imaging modalities, is essential to rule out complicating PI. This small-bowel-involved type was shown to be significantly associated with older age and chronic kidney disease in the present study. Among the four cases with chronic kidney disease, 3 were the small-bowel-only type while 1 was the large-bowel-only type, the difference of which seems marginal with *P*-values of 0.0468. There have been no reports regarding the association between affected segments of PI and kidney disease, but DuBose et al. described that patients with pathologic type were more likely to be older, with a history of enteritis and chronic renal failure [6]. Chronic kidney disease, especially end-stage renal disease increased intestinal permeability[11], which might be associated with PI affecting both the small and large bowels. In contrast to the large-bowel-only type associated with a-glucosidase inhibitors and ulcerative colitis, the small-bowel-involved type associated with older age, autoimmune disease, chronic kidney disease, and cancer can be more intractable and vulnerable to blood perfusion, which leads to the speculation of this type more complicating.

Regarding treatment, oxygen therapy was significantly associated with patients with a past medical history of bowel obstruction, and surgery was significantly associated with complicating PI. Hyperbaric oxygen therapy is a controversial treatment for adhesive postoperative small bowel obstruction, but Fukami et al. described that 143 patients (87.7%) were treated successfully with hyperbaric oxygen therapy without long-tube decompression. This oxygen therapy was associated with earlier resumption of oral intake and a shorter hospital stay, and the rate of operation was 7.4% in the hyperbaric oxygen therapy group and 14.8% in group treated by decompression alone [12]. In this context, patients with PI with a history of bowel obstruction likely underwent oxygen therapy. Duron et al. reported that abdominal distention, peritonitis, and lactic acidemia were predictive of positive intraoperative findings mandating intervention including mesenteric ischemia, an obstruction, or an incarcerated hernia on multivariate analysis in a retrospective multicenter record review of 150 PI patients, 54 (36%) of whom were managed nonoperatively, 72 of whom underwent surgery, and 24 of whom were given comfort measures only [13]. Generally, complicating or pathologic PI is an indication for surgery, as shown in the present study.

The last finding of the present study was that complicating PI was significantly associated with exacerbation of PI and subsequent death, which also makes medical sense. Wiesner et al. reported that of seven patients with infarction limited to one bowel segment (jejunum, ileum, or colon), only one patient (14%) died, whereas of the 10 patients with infarction of two or three bowel segments, eight patients (80%) died. These authors concluded that CT findings of PI and portomesenteric venous gas due to bowel ischemia do not generally allow prediction of transmural bowel infarction because these findings may be observed in patients with only partial ischemic bowel wall damage, and the clinical outcomes of patients with bowel ischemia with these CT findings seem to depend mainly on the severity and extent of their underlying disease [14], which is consistent with our comprehensive finding.

The present study has inherent limitations, including its retrospective design, ethnically homogeneous sample, low response rate to this survey, no laboratory data or images included, no CKD classification obtained, and participation bias in terms of data collection, which was conducted mainly by gastroenterologists and a few surgeons but no radiologists or acute care physicians. Therefore, the proportion of surgery in the treatment was as low as 4.6%.

## Conclusions

In conclusion, our study highlights that small-bowel-involved type and symptomatic PI are associated with complications which are indicators of poor prognosis in the largest Asian population ever.

## Supplementary Information

**Additional file 1**. Supplementary Table 1.

**Additional file 2**. Supplementary Table 2.

**Additional file 3**. Supplementary Table 3.

**Additional file 4**. Supplementary Table 4.

**Additional file 5**. Supplementary Table 5.

**Additional file 6**. Supplementary Table 6.

## Data Availability

All data generated or analysed during this study are included in this article and its supplementary material tables. Further enquiries can be directed to the corresponding author.

## Abbreviation

PI: Pneumatosis intestinalis
