# Supplementary material for: Multicenter epidemiological survey of pneumatosis intestinalis in Japan
Source: BMC Gastroenterol. 2022 May 31;22:272. doi: 10.1186/s12876-022-02343-5 (PMC9153137; doi:10.1186/s12876-022-02343-5)
Supplement: Supplementary file 1 — Additional file 1. Supplementary Table 1. [file 12876_2022_2343_MOESM1_ESM.docx]

| Supplementary Table 1. Survey parameters | | |
| --- | --- | --- |
| 1 | Gender |  |
| 2 | Age of onset |  |
| 3 | Exposure to organic solvents | Trichloroethylene or others |
| 4 | Medication used | α-glucosidase inhibitors, corticosteroid, anti-cancer agents, or others |
| 5 | Comorbidities or past history | Gastrointestinal disease, diabetes mellitus, collagen diseases, chronic lung disease, or others |
| 6 | Symptom | None, abdominal distention, pain, melena, hematochezia, diarrhea, or constipation |
| 7 | Diagnostic examination | Plain abdominal roentgenogram, computed tomography, Barium enema roentgenogram, endoscopy, endoscopic ultrasonography, laparotomy |
| 8 | Location | Large bowel (right-, left-sided colon, rectum), small bowel (duodenum, jejunum, ileum), stomach, esophagus, and others |
| 9 | Complications | Transmural ischemia, strangulation, bowel obstruction, adyamic ileus without mechanical obstruction, sepsis, shock, and massive gastrointestinal bleeding requiring blood transfusion |
| 10 | Treatment | Medical, oxygen therapy (hyperbaric and conventional), endoscopic therapy, surgery |
| 11 | Outcome | Improvement, no change, recurrence, death |
