# Supplementary material for: Multicenter epidemiological survey of pneumatosis intestinalis in Japan
Source: BMC Gastroenterol. 2022 May 31;22:272. doi: 10.1186/s12876-022-02343-5 (PMC9153137; doi:10.1186/s12876-022-02343-5)
Supplement: Supplementary file 2 — Additional file 2. Supplementary Table 2. [file 12876_2022_2343_MOESM2_ESM.docx]

| Supplementary Table 2. Segment of bowel involved | | | | | | | | | | | | | | | | | |
| --- | --- | --- | --- | --- | --- | --- | --- | --- | --- | --- | --- | --- | --- | --- | --- | --- | --- |
|  |  |  |  | Large-bowel-only type | | | | |  | Small-bowel-involved type | | | | |  | Crude  OR | *P* |
|  |  |  |  | n | | ( | % | ) |  | n | | ( | % | ) |  |  |  |
| Characteristics | | |  | Presence | Absence |  |  |  |  | Presence | Absence |  |  |  |  |  |  |
| No.patients | |  |  | 119 | | ( | 40.1 | ) |  | 46 | | ( | 15.5 | ) |  |  |  |
| Men/women | |  |  | 64/55 | | ( | 1.5 | ) |  | 23/23 | | ( | 1.0 | ) |  | 0.86 | 0.2999 |
| Median age of onset (y) | | |  | 63 (range 9-87) | | | | |  | 69 (range 47-91) | |  |  |  |  |  | 0.0010 |
| Exposure to organic solvents | | | | 2 | 117 | ( | 1.7 | ) |  | 0 | 46 | ( | 0.0 | ) |  | 0.00 | > 0.9999 |
| Medications used | | |  | 92 | 27 | ( | 77.3 | ) |  | 43 | 3 | ( | 93.5 | ) |  | 4.21 | 0.0285 |
|  | Corticosteroid | |  | 34 | 85 | ( | 21.5 | ) |  | 11 | 32 | ( | 25.6 | ) |  | 0.86 | 0.7075 |
|  | Antidiabetics | |  | 23 | 96 | ( | 14.6 | ) |  | 6 | 37 | ( | 14.0 | ) |  | 0.68 | 0.4308 |
|  |  | α-glucosidase inhibitors | | 28 | 91 | ( | 17.7 | ) |  | 4 | 39 | ( | 9.3 | ) |  | 0.33 | 0.0469 |
|  |  | Sulfonylurea | | 5 | 114 | ( | 3.2 | ) |  | 1 | 42 | ( | 2.3 | ) |  | 0.54 | > 0.9999 |
|  |  | Glinide |  | 3 | 116 | ( | 1.9 | ) |  | 0 | 43 | ( | 0.0 | ) |  | 0.00 | 0.5659 |
|  |  | Insulin |  | 2 | 117 | ( | 1.3 | ) |  | 1 | 42 | ( | 2.3 | ) |  | 1.39 | > 0.9999 |
|  |  | Biguanide |  | 1 | 118 | ( | 0.6 | ) |  | 0 | 43 | ( | 0.0 | ) |  | 0.00 | > 0.9999 |
|  |  | Dipeptidyl peptidase 4 inhibitor | | 1 | 118 | ( | 0.6 | ) |  | 0 | 43 | ( | 0.0 | ) |  | 0.00 | > 0.9999 |
|  | Immunosuppressants | | | 10 | 109 | ( | 6.3 | ) |  | 6 | 37 | ( | 14.0 | ) |  | 1.77 | 0.2958 |
|  | Anti-cancer agents | | | 5 | 114 | ( | 3.2 | ) |  | 4 | 39 | ( | 9.3 | ) |  | 2.34 | 0.2743 |
|  | Antihypertensives | | | 16 | 103 | ( | 10.1 | ) |  | 3 | 40 | ( | 7.0 | ) |  | 0.48 | 0.4067 |
|  |  | Calcium antagonist | | 9 | 110 | ( | 5.7 | ) |  | 1 | 42 | ( | 2.3 | ) |  | 0.29 | 0.2927 |
|  |  | β-blocker |  | 8 | 111 | ( | 5.1 | ) |  | 0 | 43 | ( | 0.0 | ) |  | 0.00 | 0.1109 |
|  |  | Angiotensin II receptor blocker | | 7 | 112 | ( | 4.4 | ) |  | 2 | 41 | ( | 4.7 | ) |  | 0.78 | > 0.9999 |
|  |  | Angiotensin converting enzyme inhibitor | | 1 | 118 | ( | 0.6 | ) |  | 0 | 43 | ( | 0.0 | ) |  | 0.00 | > 0.9999 |
|  |  | α-blocker |  | 0 | 119 | ( | 0.0 | ) |  | 1 | 42 | ( | 2.3 | ) |  | n.d | 0.2654 |
|  | Diuretics | |  | 2 | 117 | ( | 1.3 | ) |  | 3 | 40 | ( | 7.0 | ) |  | 4.39 | 0.1171 |
|  | Digitalis | |  | 3 | 116 | ( | 1.9 | ) |  | 1 | 42 | ( | 2.3 | ) |  | 0.92 | > 0.9999 |
|  | Antiarrythmics | |  | 1 | 118 | ( | 0.6 | ) |  | 1 | 42 | ( | 2.3 | ) |  | 2.81 | 0.4616 |
|  | Antithrombotics | |  | 10 | 109 | ( | 6.3 | ) |  | 2 | 41 | ( | 4.7 | ) |  | 0.53 | 0.5190 |
|  |  | Anticoagulants | | 4 | 115 | ( | 2.5 | ) |  | 0 | 43 | ( | 0.0 | ) |  | 0.00 | 0.5741 |
|  |  | Antiplatelets | | 7 | 112 | ( | 4.4 | ) |  | 2 | 41 | ( | 4.7 | ) |  | 0.78 | > 0.9999 |
|  | Bronchodilators | |  | 3 | 116 | ( | 1.9 | ) |  | 0 | 43 | ( | 0.0 | ) |  | 0.00 | 0.5659 |
|  | Gastric acid secretion inhibitors | | | 15 | 104 | ( | 9.5 | ) |  | 5 | 38 | ( | 11.6 | ) |  | 0.91 | > 0.9999 |
|  |  | Proton pump inhibitors | | 11 | 108 | ( | 7.0 | ) |  | 4 | 39 | ( | 9.3 | ) |  | 1.01 | > 0.9999 |
|  |  | Histamine-2 receptor antagonists | | 5 | 114 | ( | 3.2 | ) |  | 1 | 42 | ( | 2.3 | ) |  | 0.54 | > 0.9999 |
|  | 5-aminosalicylates or salicylazosulfapyridine | | | 15 | 104 | ( | 9.5 | ) |  | 0 | 43 | ( | 0.0 | ) |  | 0.00 | 0.0119 |
|  | Nonsteroidal antiinflammatory drugs | | | 2 | 117 | ( | 1.3 | ) |  | 1 | 42 | ( | 2.3 | ) |  | 1.39 | > 0.9999 |
|  | Antibiotics | |  | 7 | 112 | ( | 4.4 | ) |  | 2 | 41 | ( | 4.7 | ) |  | 0.78 | > 0.9999 |
|  |  | Trimethoprim-sulfamethoxazole | | 4 | 115 | ( | 2.5 | ) |  | 2 | 41 | ( | 4.7 | ) |  | 1.40 | 0.6565 |
|  | Laxatives | |  | 5 | 114 | ( | 3.2 | ) |  | 5 | 38 | ( | 11.6 | ) |  | 3.00 | 0.1318 |
|  | Bisphophonates | |  | 2 | 117 | ( | 1.3 | ) |  | 0 | 43 | ( | 0.0 | ) |  | 0.00 | > 0.9999 |
|  | Statins / ezetimib/ fibrates | | | 14 | 105 | ( | 8.9 | ) |  | 0 | 43 | ( | 0.0 | ) |  | 0.00 | 0.0220 |
|  | Hypnotics | |  | 1 | 118 | ( | 0.6 | ) |  | 1 | 42 | ( | 2.3 | ) |  | 2.81 | 0.4616 |
|  | Psychotropics | |  | 4 | 115 | ( | 2.5 | ) |  | 2 | 41 | ( | 4.7 | ) |  | 1.40 | 0.6565 |
|  | Prostatic hypertrophy drugs | | | 2 | 117 | ( | 1.3 | ) |  | 2 | 41 | ( | 4.7 | ) |  | 2.85 | 0.2869 |
|  | Allopurinol / benzbromaron | | | 2 | 117 | ( | 1.3 | ) |  | 1 | 42 | ( | 2.3 | ) |  | 1.39 | > 0.9999 |
|  | Levothyroxine | |  | 4 | 115 | ( | 2.5 | ) |  | 0 | 43 | ( | 0.0 | ) |  | 0.00 | 0.5741 |
|  | Herbal medicine | |  | 4 | 115 | ( | 2.5 | ) |  | 3 | 40 | ( | 7.0 | ) |  | 2.16 | 0.3834 |
| Comobidities and/or past medical history | | | | 101 | 18 | ( | 84.9 | ) |  | 37 | 8 | ( | 82.2 | ) |  | 0.82 | 0.6782 |
|  | Gastroduodenal diseases | | | 36 | 83 | ( | 30.3 | ) |  | 8 | 37 | ( | 17.8 | ) |  | 0.50 | 0.1077 |
|  |  | Inflammatory bowel disease | | 15 | 104 | ( | 12.6 | ) |  | 0 | 45 | ( | 0.0 | ) |  | 0.00 | 0.0119 |
|  |  |  | Ulcerative colitis | 12 | 107 | ( | 10.1 | ) |  | 0 | 45 | ( | 0.0 | ) |  | 0.00 | 0.0377 |
|  |  |  | Crohn's disease | 2 | 117 | ( | 1.7 | ) |  | 0 | 45 | ( | 0.0 | ) |  | 0.00 | > 0.9999 |
|  |  |  | Behcet's disease | 1 | 118 | ( | 0.8 | ) |  | 0 | 45 | ( | 0.0 | ) |  | 0.00 | > 0.9999 |
|  |  | Carcinoma | | 8 | 111 | ( | 6.7 | ) |  | 5 | 40 | ( | 11.1 | ) |  | 1.73 | 0.3472 |
|  |  |  | Esophegeal carcinoma | 0 | 119 | ( | 0.0 | ) |  | 1 | 44 | ( | 2.2 | ) |  | n.d | 0.2744 |
|  |  |  | Gastric carcinoma | 1 | 118 | ( | 0.8 | ) |  | 2 | 43 | ( | 4.4 | ) |  | 5.49 | 0.1829 |
|  |  |  | Colorectal carcinoma | 7 | 112 | ( | 5.9 | ) |  | 2 | 43 | ( | 4.4 | ) |  | 0.74 | > 0.9999 |
|  |  | Colorectal polyp | | 6 | 113 | ( | 5.0 | ) |  | 0 | 45 | ( | 0.0 | ) |  | 0.00 | 0.1896 |
|  |  | Bowel obstruction | | 3 | 116 | ( | 2.5 | ) |  | 1 | 44 | ( | 2.2 | ) |  | 0.88 | > 0.9999 |
|  |  | Others |  | 8 | 111 | ( | 6.7 | ) |  | 0 | 45 | ( | 0.0 | ) |  | 0.00 | 0.1083 |
|  |  |  | Esophegeal candidiasis | 1 | 118 | ( | 0.8 | ) |  | 0 | 45 | ( | 0.0 | ) |  | 0.00 | > 0.9999 |
|  |  |  | Gastroesophageal reflux disease | 1 | 118 | ( | 0.8 | ) |  | 0 | 45 | ( | 0.0 | ) |  | 0.00 | > 0.9999 |
|  |  |  | Peptic ulcer disease | 2 | 117 | ( | 1.7 | ) |  | 0 | 45 | ( | 0.0 | ) |  | 0.00 | > 0.9999 |
|  |  |  | Ischemic colitis | 1 | 118 | ( | 0.8 | ) |  | 0 | 45 | ( | 0.0 | ) |  | 0.00 | > 0.9999 |
|  | Hepatobiliarypancreatic disease | | | 6 | 113 | ( | 5.0 | ) |  | 3 | 42 | ( | 6.7 | ) |  | 1.35 | 0.7068 |
|  |  | Hepatic hemangioma | | 1 | 118 | ( | 0.8 | ) |  | 0 | 45 | ( | 0.0 | ) |  | 0.00 | > 0.9999 |
|  |  | Chronic hepatitis | | 1 | 118 | ( | 0.8 | ) |  | 0 | 45 | ( | 0.0 | ) |  | 0.00 | > 0.9999 |
|  |  | Cirrhosis |  | 0 | 119 | ( | 0.0 | ) |  | 1 | 44 | ( | 2.2 | ) |  | n.d | 0.2744 |
|  |  | Cholecystitis | | 3 | 116 | ( | 2.5 | ) |  | 1 | 44 | ( | 2.2 | ) |  | 0.88 | > 0.9999 |
|  |  | Chroinc pancreatitis | | 0 | 119 | ( | 0.0 | ) |  | 1 | 44 | ( | 2.2 | ) |  | n.d | 0.2744 |
|  | Diabetes mellitus | |  | 21 | 98 | ( | 17.6 | ) |  | 8 | 37 | ( | 17.8 | ) |  | 1.01 | > 0.9999 |
|  | Chronic lung disease | | | 18 | 101 | ( | 15.1 | ) |  | 10 | 35 | ( | 22.2 | ) |  | 1.60 | 0.2812 |
|  | Autoimmune disease | | | 16 | 103 | ( | 13.4 | ) |  | 12 | 33 | ( | 26.7 | ) |  | 2.34 | 0.0447 |
|  | Hypertension | |  | 10 | 109 | ( | 8.4 | ) |  | 3 | 42 | ( | 6.7 | ) |  | 0.78 | > 0.9999 |
|  | Heart disease | |  | 8 | 111 | ( | 6.7 | ) |  | 3 | 42 | ( | 6.7 | ) |  | 0.99 | > 0.9999 |
|  | Dyslipidemia | |  | 8 | 111 | ( | 6.7 | ) |  | 1 | 44 | ( | 2.2 | ) |  | 0.32 | 0.4465 |
|  | Hematological disease | | | 7 | 112 | ( | 5.9 | ) |  | 0 | 45 | ( | 0.0 | ) |  | 0.00 | 0.1914 |
|  |  | Bone marrow transplantation | | 3 | 116 | ( | 2.5 | ) |  | 0 | 45 | ( | 0.0 | ) |  | 0.00 | 0.5623 |
|  | Kideny disease | |  | 1 | 118 | ( | 0.8 | ) |  | 3 | 42 | ( | 6.7 | ) |  | 8.43 | 0.0633 |
|  | Hyperuricemia | |  | 3 | 116 | ( | 2.5 | ) |  | 0 | 45 | ( | 0.0 | ) |  | 0.00 | 0.5623 |
|  | Psychiatric diseases | | | 3 | 116 | ( | 2.5 | ) |  | 1 | 44 | ( | 2.2 | ) |  | 0.88 | > 0.9999 |
|  | Neurological diseases | | | 4 | 115 | ( | 3.4 | ) |  | 0 | 45 | ( | 0.0 | ) |  | 0.00 | 0.5759 |
|  | Peripheral vascular disease | | | 1 | 118 | ( | 0.8 | ) |  | 0 | 45 | ( | 0.0 | ) |  | 0.00 | > 0.9999 |
|  | Endocine disease | | | 4 | 115 | ( | 3.4 | ) |  | 0 | 45 | ( | 0.0 | ) |  | 0.00 | 0.5759 |
|  | Cancer except the digestive or hematologic system | | | 2 | 117 | ( | 1.7 | ) |  | 8 | 37 | ( | 17.8 | ) |  | 12.65 | 0.0006 |
