# Supplementary material for: Multicenter epidemiological survey of pneumatosis intestinalis in Japan
Source: BMC Gastroenterol. 2022 May 31;22:272. doi: 10.1186/s12876-022-02343-5 (PMC9153137; doi:10.1186/s12876-022-02343-5)
Supplement: Supplementary file 3 — Additional file 3. Supplementary Table 3. [file 12876_2022_2343_MOESM3_ESM.docx]

| Supplementary Table 3. Complicating pneumatosis intestinalis | | | | | | | | | | | | | | | | | |
| --- | --- | --- | --- | --- | --- | --- | --- | --- | --- | --- | --- | --- | --- | --- | --- | --- | --- |
|  |  |  |  | Benign | | | | |  | Complicating | | | | |  | Crude  OR | *P* |
|  |  |  |  | n | | ( | % | ) |  | n | | ( | % | ) |  |  |  |
| Characteristics | |  |  | Presence | Absence |  |  |  |  | Presence | Absence |  |  |  |  |  |  |
| No.patients | |  |  | 141 | | ( | 84.4 | ) |  | 26 | | ( | 15.6 | ) |  |  |  |
| Men/women | |  |  | 74/67 | | ( | 1.4 | ) |  | 13/13 | | ( | 1.0 | ) |  | 0.91 | 0.8159 |
| Median age of onset (y) | |  |  | 64 (range 9-87) | | | | |  | 68 (range 9-91) | |  |  |  |  |  | 0.8159 |
| Symptoms | |  |  | 72 | 69 | ( | 51.1 | ) |  | 25 | 1 | ( | 96.2 | ) |  | 23.96 | < 0.0001 |
| Exposure to organic solvents | |  |  | 2 | 139 | ( | 1.4 | ) |  | 0 | 26 | ( | 0.0 | ) |  | 0.00 | > 0.9999 |
| Medications used | | |  | 102 | 39 | ( | 72.3 | ) |  | 20 | 6 | ( | 76.9 | ) |  | 1.27 | 0.6284 |
|  | Corticosteroid |  |  | 39 | 102 | ( | 27.7 | ) |  | 6 | 15 | ( | 28.6 | ) |  | 1.05 | 0.9306 |
|  | Antidiabetics |  |  | 25 | 116 | ( | 17.7 | ) |  | 4 | 17 | ( | 19.0 | ) |  | 1.09 | > 0.9999 |
|  |  | α-glucosidase inhibitors | | 20 | 121 | ( | 14.2 | ) |  | 3 | 18 | ( | 14.3 | ) |  | 1.01 | > 0.9999 |
|  |  | Sulfonylurea |  | 6 | 135 | ( | 4.3 | ) |  | 0 | 21 | ( | 0.0 | ) |  | 0.00 | > 0.9999 |
|  |  | Glinide |  | 3 | 138 | ( | 2.1 | ) |  | 0 | 21 | ( | 0.0 | ) |  | 0.00 | > 0.9999 |
|  |  | Insulin |  | 2 | 139 | ( | 1.4 | ) |  | 1 | 20 | ( | 4.8 | ) |  | 3.48 | 0.3245 |
|  |  | Biguanide |  | 1 | 140 | ( | 0.7 | ) |  | 0 | 21 | ( | 0.0 | ) |  | 0.00 | > 0.9999 |
|  |  | Dipeptidyl peptidase 4 inhibitor | | 1 | 140 | ( | 0.7 | ) |  | 0 | 21 | ( | 0.0 | ) |  | 0.00 | > 0.9999 |
|  | Immunosuppressants | |  | 12 | 129 | ( | 8.5 | ) |  | 4 | 17 | ( | 19.0 | ) |  | 2.53 | 0.1332 |
|  | Anti-cancer agents |  |  | 7 | 134 | ( | 5.0 | ) |  | 2 | 19 | ( | 9.5 | ) |  | 2.02 | 0.3295 |
|  | Antihypertensives |  |  | 16 | 125 | ( | 11.3 | ) |  | 3 | 18 | ( | 14.3 | ) |  | 1.30 | 0.7163 |
|  |  | Calcium antagonist | | 10 | 131 | ( | 7.1 | ) |  | 0 | 21 | ( | 0.0 | ) |  | 0.00 | 0.3626 |
|  |  | β-blocker |  | 7 | 134 | ( | 5.0 | ) |  | 1 | 20 | ( | 4.8 | ) |  | 0.96 | > 0.9999 |
|  |  | Angiotensin II receptor blocker | | 13 | 128 | ( | 9.2 | ) |  | 1 | 20 | ( | 4.8 | ) |  | 0.49 | 0.6968 |
|  |  | Angiotensin converting enzyme inhibitor | | 1 | 140 | ( | 0.7 | ) |  | 0 | 21 | ( | 0.0 | ) |  | 0.00 | > 0.9999 |
|  |  | α-blocker |  | 0 | 141 | ( | 0.0 | ) |  | 1 | 20 | ( | 4.8 | ) |  | n.d | 0.1296 |
|  | Diuretics |  |  | 3 | 138 | ( | 2.1 | ) |  | 2 | 19 | ( | 9.5 | ) |  | 4.84 | 0.1259 |
|  | Digitalis |  |  | 4 | 137 | ( | 2.8 | ) |  | 0 | 21 | ( | 0.0 | ) |  | 0.00 | > 0.9999 |
|  | Antiarrythmics |  |  | 1 | 140 | ( | 0.7 | ) |  | 1 | 20 | ( | 4.8 | ) |  | 7.00 | 0.2432 |
|  | Antithrombotics |  |  | 10 | 131 | ( | 7.1 | ) |  | 2 | 19 | ( | 9.5 | ) |  | 1.38 | 0.6563 |
|  |  | Anticoagulants |  | 4 | 137 | ( | 2.8 | ) |  | 0 | 21 | ( | 0.0 | ) |  | 0.00 | > 0.9999 |
|  |  | Antiplatelets |  | 7 | 134 | ( | 5.0 | ) |  | 2 | 19 | ( | 9.5 | ) |  | 2.02 | 0.3295 |
|  | Bronchodilators |  |  | 3 | 138 | ( | 2.1 | ) |  | 0 | 21 | ( | 0.0 | ) |  | 0.00 | > 0.9999 |
|  | Gastric acid secretion inhibitors | |  | 17 | 124 | ( | 12.1 | ) |  | 3 | 18 | ( | 14.3 | ) |  | 1.22 | 0.7276 |
|  |  | Proton pump inhibitors | | 12 | 129 | ( | 8.5 | ) |  | 3 | 18 | ( | 14.3 | ) |  | 1.79 | 0.4163 |
|  |  | Histamine-2 receptor antagonists | | 6 | 135 | ( | 4.3 | ) |  | 0 | 21 | ( | 0.0 | ) |  | 0.00 | > 0.9999 |
|  | 5-aminosalicylates or salicylazosulfapyridine | | | 14 | 127 | ( | 9.9 | ) |  | 1 | 20 | ( | 4.8 | ) |  | 0.45 | 0.6951 |
|  | Nonsteroidal antiinflammatory drugs | |  | 2 | 139 | ( | 1.4 | ) |  | 1 | 20 | ( | 4.8 | ) |  | 3.48 | 0.3245 |
|  | Antibiotics |  |  | 7 | 134 | ( | 5.0 | ) |  | 2 | 19 | ( | 9.5 | ) |  | 2.02 | 0.3295 |
|  |  | Trimethoprim-sulfamethoxazole | | 4 | 137 | ( | 2.8 | ) |  | 2 | 19 | ( | 9.5 | ) |  | 3.61 | 0.1743 |
|  | Laxatives |  |  | 5 | 136 | ( | 3.5 | ) |  | 5 | 16 | ( | 23.8 | ) |  | 8.50 | 0.0038 |
|  | Bisphophonates |  |  | 2 | 139 | ( | 1.4 | ) |  | 0 | 21 | ( | 0.0 | ) |  | 0.00 | > 0.9999 |
|  | Statins / ezetimib/ fibrates | |  | 13 | 128 | ( | 9.2 | ) |  | 2 | 19 | ( | 9.5 | ) |  | 1.04 | > 0.9999 |
|  | Hypnotics |  |  | 2 | 139 | ( | 1.4 | ) |  | 0 | 21 | ( | 0.0 | ) |  | 0.00 | > 0.9999 |
|  | Psychotropics |  |  | 5 | 136 | ( | 3.5 | ) |  | 1 | 20 | ( | 4.8 | ) |  | 1.36 | 0.5714 |
|  | Prostatic hypertrophy drugs | |  | 2 | 139 | ( | 1.4 | ) |  | 2 | 19 | ( | 9.5 | ) |  | 7.32 | 0.0820 |
|  | Allopurinol / benzbromaron | |  | 1 | 140 | ( | 0.7 | ) |  | 2 | 19 | ( | 9.5 | ) |  | 14.74 | 0.0445 |
|  | Levothyroxine |  |  | 4 | 137 | ( | 2.8 | ) |  | 0 | 21 | ( | 0.0 | ) |  | 0.00 | > 0.9999 |
|  | Herbal medicine |  |  | 4 | 137 | ( | 2.8 | ) |  | 3 | 18 | ( | 14.3 | ) |  | 5.71 | 0.0470 |
| Comobidities and/or past medical history | | | | 116 | 25 | ( | 82.3 | ) |  | 24 | 2 | ( | 92.3 | ) |  | 2.59 | 0.2014 |
|  | Gastrointestinal diseases | |  | 41 | 100 | ( | 29.1 | ) |  | 4 | 22 | ( | 15.4 | ) |  | 0.44 | 0.2278 |
|  |  | Inflammatory bowel disease | | 14 | 127 | ( | 9.9 | ) |  | 2 | 24 | ( | 7.7 | ) |  | 0.76 | > 0.9999 |
|  |  |  | Ulcerative colitis | 11 | 130 | ( | 7.8 | ) |  | 2 | 24 | ( | 7.7 | ) |  | 0.98 | > 0.9999 |
|  |  |  | Crohn's disease | 2 | 139 | ( | 1.4 | ) |  | 0 | 26 | ( | 0.0 | ) |  | 0.00 | > 0.9999 |
|  |  |  | Behcet's disease | 1 | 140 | ( | 0.7 | ) |  | 0 | 26 | ( | 0.0 | ) |  | 0.00 | > 0.9999 |
|  |  | Carcinoma |  | 12 | 129 | ( | 8.5 | ) |  | 1 | 25 | ( | 3.8 | ) |  | 0.43 | 0.6942 |
|  |  |  | Esophegeal carcinoma | 1 | 140 | ( | 0.7 | ) |  | 0 | 26 | ( | 0.0 | ) |  | 0.00 | > 0.9999 |
|  |  |  | Gastric carcinoma | 3 | 138 | ( | 2.1 | ) |  | 0 | 26 | ( | 0.0 | ) |  | 0.00 | > 0.9999 |
|  |  |  | Colorectal carcinoma | 8 | 133 | ( | 5.7 | ) |  | 1 | 25 | ( | 3.8 | ) |  | 0.67 | > 0.9999 |
|  |  | Colorectal polyp |  | 6 | 135 | ( | 4.3 | ) |  | 0 | 26 | ( | 0.0 | ) |  | 0.00 | 0.5915 |
|  |  | Bowel obstruction |  | 4 | 137 | ( | 2.8 | ) |  | 0 | 26 | ( | 0.0 | ) |  | 0.00 | > 0.9999 |
|  |  | Others |  | 5 | 136 | ( | 3.5 | ) |  | 0 | 26 | ( | 0.0 | ) |  | 0.00 | > 0.9999 |
|  |  |  | Esophegeal candidiasis | 1 | 140 | ( | 0.7 | ) |  | 0 | 26 | ( | 0.0 | ) |  | 0.00 | > 0.9999 |
|  |  |  | Gastroesophageal reflux disease | 1 | 140 | ( | 0.7 | ) |  | 0 | 26 | ( | 0.0 | ) |  | 0.00 | > 0.9999 |
|  |  |  | Peptic ulcer disease | 2 | 139 | ( | 1.4 | ) |  | 0 | 26 | ( | 0.0 | ) |  | 0.00 | > 0.9999 |
|  |  |  | Ischemic colitis | 1 | 140 | ( | 0.7 | ) |  | 0 | 26 | ( | 0.0 | ) |  | 0.00 | > 0.9999 |
|  | Hepatobiliarypancreatic disease | |  | 9 | 132 | ( | 6.4 | ) |  | 1 | 25 | ( | 3.8 | ) |  | 0.59 | > 0.9999 |
|  |  | Hepatic hemangioma | | 1 | 140 | ( | 0.7 | ) |  | 0 | 26 | ( | 0.0 | ) |  | 0.00 | > 0.9999 |
|  |  | Chronic hepatitis |  | 2 | 139 | ( | 1.4 | ) |  | 0 | 26 | ( | 0.0 | ) |  | 0.00 | > 0.9999 |
|  |  | Cirrhosis |  | 1 | 140 | ( | 0.7 | ) |  | 0 | 26 | ( | 0.0 | ) |  | 0.00 | > 0.9999 |
|  |  | Hepatic carcinoma | | 1 | 140 | ( | 0.7 | ) |  | 1 | 25 | ( | 3.8 | ) |  | 5.60 | 0.2879 |
|  |  | Cholecystitis |  | 4 | 137 | ( | 2.8 | ) |  | 0 | 26 | ( | 0.0 | ) |  | 0.00 | > 0.9999 |
|  |  | Chroinc pancreatitis | | 1 | 140 | ( | 0.7 | ) |  | 0 | 26 | ( | 0.0 | ) |  | 0.00 | > 0.9999 |
|  | Diabetes mellitus |  |  | 23 | 118 | ( | 16.3 | ) |  | 8 | 18 | ( | 30.8 | ) |  | 2.28 | 0.0815 |
|  | Chronic lung disease | |  | 24 | 117 | ( | 17.0 | ) |  | 4 | 22 | ( | 15.4 | ) |  | 0.89 | > 0.9999 |
|  | Autoimmune disease | |  | 25 | 116 | ( | 17.7 | ) |  | 6 | 20 | ( | 23.1 | ) |  | 1.39 | 0.5194 |
|  |  | Rheumatoid arthritis | | 9 | 132 | ( | 6.4 | ) |  | 2 | 24 | ( | 7.7 | ) |  | 1.22 | 0.6816 |
|  |  | Systemic sclerosis | | 7 | 134 | ( | 5.0 | ) |  | 2 | 24 | ( | 7.7 | ) |  | 1.60 | 0.6319 |
|  |  | Dermatomyositis / polymyositis | | 4 | 137 | ( | 2.8 | ) |  | 1 | 25 | ( | 3.8 | ) |  | 1.37 | 0.5758 |
|  |  | Systemic lupus erythematosus | | 4 | 137 | ( | 2.8 | ) |  | 1 | 25 | ( | 3.8 | ) |  | 1.37 | 0.5758 |
|  | Hypertension |  |  | 10 | 131 | ( | 7.1 | ) |  | 3 | 23 | ( | 11.5 | ) |  | 1.71 | 0.4292 |
|  | Heart disease |  |  | 8 | 133 | ( | 5.7 | ) |  | 2 | 24 | ( | 7.7 | ) |  | 1.39 | 0.6557 |
|  | Dyslipidemia |  |  | 9 | 132 | ( | 6.4 | ) |  | 0 | 26 | ( | 0.0 | ) |  | 0.00 | 0.3572 |
|  | Hematological disease | |  | 7 | 134 | ( | 5.0 | ) |  | 1 | 25 | ( | 3.8 | ) |  | 0.77 | > 0.9999 |
|  |  | Bone marrow transplantation | | 2 | 139 | ( | 1.4 | ) |  | 0 | 26 | ( | 0.0 | ) |  | 0.00 | > 0.9999 |
|  | Kideny disease |  |  | 3 | 138 | ( | 2.1 | ) |  | 2 | 24 | ( | 7.7 | ) |  | 3.83 | 0.1732 |
|  | Hyperuricemia |  |  | 1 | 140 | ( | 0.7 | ) |  | 1 | 25 | ( | 3.8 | ) |  | 5.60 | 0.2879 |
|  | Psychiatric diseases | |  | 3 | 138 | ( | 2.1 | ) |  | 1 | 25 | ( | 3.8 | ) |  | 1.84 | 0.4952 |
|  | Neurological diseases | |  | 4 | 137 | ( | 2.8 | ) |  | 2 | 24 | ( | 7.7 | ) |  | 2.85 | 0.2353 |
|  | Peripheral vascular disease | |  | 1 | 140 | ( | 0.7 | ) |  | 0 | 26 | ( | 0.0 | ) |  | 0.00 | > 0.9999 |
|  | Endocine disease |  |  | 4 | 137 | ( | 2.8 | ) |  | 0 | 26 | ( | 0.0 | ) |  | 0.00 | > 0.9999 |
|  | Cancer except the digestive or hematologic system | | | 7 | 134 | ( | 5.0 | ) |  | 3 | 23 | ( | 11.5 | ) |  | 2.50 | 0.1897 |
| Segments involved | |  |  |  |  |  |  |  |  |  |  |  |  |  |  |  |  |
|  | Large bowel only |  |  | 112 | 29 | ( | 79.4 | ) |  | 7 | 19 | ( | 26.92307692 | ) |  | 0.10 | < 0.0001 |
|  |  | Right-sided colon only | | 81 | 60 | ( | 57.4 | ) |  | 2 | 24 | ( | 7.7 | ) |  | 0.06 | < 0.0001 |
|  |  | Left-sided colon only | | 22 | 119 | ( | 15.6 | ) |  | 4 | 22 | ( | 15.4 | ) |  | 0.98 | > 0.9999 |
|  |  | Rectum only |  | 1 | 140 | ( | 0.7 | ) |  | 0 | 26 | ( | 0.0 | ) |  | 0.00 | > 0.9999 |
|  |  | Righ- and left-sided colon | | 5 | 136 | ( | 3.5 | ) |  | 1 | 25 | ( | 3.8 | ) |  | 1.09 | > 0.9999 |
|  |  | Left-sided colon and rectum | | 1 | 140 | ( | 0.7 | ) |  | 0 | 26 | ( | 0.0 | ) |  | 0.00 | > 0.9999 |
|  |  | Throughout the large bowel | | 2 | 139 | ( | 1.4 | ) |  | 0 | 26 | ( | 0.0 | ) |  | 0.00 | > 0.9999 |
|  | Small bowel only |  |  | 24 | 117 | ( | 17.0 | ) |  | 9 | 17 | ( | 34.6 | ) |  | 2.58 | 0.0384 |
|  |  | Ileum only |  | 8 | 133 | ( | 5.7 | ) |  | 6 | 20 | ( | 23.1 | ) |  | 4.99 | 0.0033 |
|  |  | Jejunum only |  | 9 | 132 | ( | 6.4 | ) |  | 2 | 24 | ( | 7.7 | ) |  | 1.22 | 0.6816 |
|  |  | Ileum and jejunum |  | 7 | 134 | ( | 5.0 | ) |  | 1 | 25 | ( | 3.8 | ) |  | 0.77 | > 0.9999 |
|  | Combined |  |  | 5 | 136 | ( | 3.5 | ) |  | 8 | 18 | ( | 30.8 | ) |  | 12.09 | < 0.0001 |
|  |  | Ileum and right-sided colon | | 3 | 138 | ( | 2.1 | ) |  | 0 | 26 | ( | 0.0 | ) |  | 0.00 | > 0.9999 |
|  |  | Jejunum and right-sided colon | | 1 | 140 | ( | 0.7 | ) |  | 0 | 26 | ( | 0.0 | ) |  | n.d | > 0.9999 |
|  |  | Ileum, right- and left-sided colon | | 0 | 141 | ( | 0.0 | ) |  | 3 | 23 | ( | 11.5 | ) |  | n.d | 0.0034 |
|  |  | Jejunum, ileum, right- and left-sided colon | | 0 | 141 | ( | 0.0 | ) |  | 3 | 23 | ( | 11.5 | ) |  | n.d | 0.0034 |
|  |  | Esophagus, stomach, small bowel | | 1 | 140 | ( | 0.7 | ) |  | 1 | 25 | ( | 3.8 | ) |  | 5.60 | 0.2879 |
|  |  | Esophagus, stomach, small bowel, and colon | | 0 | 141 | ( | 0.0 | ) |  | 1 | 25 | ( | 3.8 | ) |  | n.d | 0.1296 |
| Treatment | |  |  |  |  |  |  |  |  |  |  |  |  |  |  |  |  |
|  | Medical treatment or observation | |  | 106 | 35 | ( | 75.2 | ) |  | 11 | 15 | ( | 42.3 | ) |  | 0.24 | 0.0008 |
|  | Oxygene therapy |  |  | 28 | 113 | ( | 19.9 | ) |  | 7 | 19 | ( | 26.9 | ) |  | 1.49 | 0.4161 |
|  |  | Hyperbaric |  | 12 | 129 | ( | 8.5 | ) |  | 4 | 22 | ( | 15.4 | ) |  | 1.95 | 0.2803 |
|  |  | Conventional |  | 16 | 125 | ( | 11.3 | ) |  | 3 | 23 | ( | 11.5 | ) |  | 1.02 | > 0.9999 |
|  | Endoscopic therapy | |  | 3 | 138 | ( | 2.1 | ) |  | 0 | 26 | ( | 0.0 | ) |  | 0.00 | > 0.9999 |
|  | Surgery |  |  | 4 | 137 | ( | 2.8 | ) |  | 8 | 18 | ( | 30.8 | ) |  | 15.22 | < 0.0001 |
| Outcome |  |  |  |  |  |  |  |  |  |  |  |  |  |  |  |  |  |
|  | Pneumatosi intestinalis | |  |  |  |  |  |  |  |  |  |  |  |  |  |  |  |
|  |  | Improvement |  | 99 | 42 | ( | 70.2 | ) |  | 20 | 6 | ( | 76.9 | ) |  | 1.41 | 0.4872 |
|  |  | No change |  | 38 | 103 | ( | 27.0 | ) |  | 2 | 24 | ( | 7.7 | ) |  | 0.23 | 0.0436 |
|  |  | Exacerbation |  | 4 | 137 | ( | 2.8 | ) |  | 4 | 22 | ( | 15.4 | ) |  | 6.23 | 0.0212 |
|  |  | Recurrence |  | 1 | 140 | ( | 0.7 | ) |  | 0 | 26 | ( | 0.0 | ) |  | 0.00 | > 0.9999 |
|  | Complicating death |  |  | 2 | 139 | ( | 1.4 | ) |  | 6 | 20 | ( | 23.1 | ) |  | 20.85 | 0.0002 |
