# Supplementary material for: Multicenter epidemiological survey of pneumatosis intestinalis in Japan
Source: BMC Gastroenterol. 2022 May 31;22:272. doi: 10.1186/s12876-022-02343-5 (PMC9153137; doi:10.1186/s12876-022-02343-5)
Supplement: Supplementary file 4 — Additional file 4. Supplementary Table 4. [file 12876_2022_2343_MOESM4_ESM.docx]

| Supplementary Table 4. Treatment of pneumatosis intestinalis | | | | | | | | | | | | | | | | | | | | | | | | | | | | |
| --- | --- | --- | --- | --- | --- | --- | --- | --- | --- | --- | --- | --- | --- | --- | --- | --- | --- | --- | --- | --- | --- | --- | --- | --- | --- | --- | --- | --- |
|  |  |  |  | Medical | | | |  | Oxygen therapy | | | | | |  | Endoscopic therapy | | | | | |  | Surgery | | | | | |
| Characteristics | |  |  | n | ( | % | ) |  | n | ( | % | ) | Crude  OR | *P* |  | n | ( | % | ) | Crude  OR | *P* |  | n | ( | % | ) | Crude  OR | *P* |
| No.patients | |  |  | 117 | ( | 70.1 | ) |  | 35 | ( | 21.0 | ) |  |  |  | 3 | ( | 1.8 | ) |  |  |  | 12 | ( | 7.2 | ) |  |  |
| Men/women | |  |  | 63/54 | ( | 1.2 | ) |  | 17/18 | ( | 0.9 | ) | 0.8 |  |  | 2/1 | ( | 2.0 | ) | 1.7 | > 0.9999 |  | 5/7 | ( | 0.7 | ) | 0.6 | 0.2699 |
| Median age of onset (y) | |  |  | 64 (range 9-91) | | | |  | 68 (range 22-80) | | | | | 0.3264 |  | 55 (range 52-59) | | | | | 0.1259 |  | 73.5 (range 19-83) | | | | | 0.0967 |
| Exposure to organic solvents | | |  | 2 | ( | 1.7 | ) |  | 0 | ( | 0.0 | ) | 0.0 | > 0.9999 |  | 0 | ( | 0.0 | ) | 0.0 | > 0.9999 |  | 0 | ( | 0.0 | ) | 0.0 | 0.4975 |
| Medications used | | |  | 89 | ( | 76.1 | ) |  | 22 | ( | 62.9 | ) | 0.5 | 0.1223 |  | 2 | ( | 66.7 | ) | 0.6 | 0.3909 |  | 9 | ( | 100.0 | ) | n.d | 0.2054 |
|  | Corticosteroid |  |  | 31 | ( | 26.5 | ) |  | 12 | ( | 34.3 | ) | 1.4 | 0.3693 |  | 0 | ( | 0.0 | ) | 0.0 | 0.1930 |  | 2 | ( | 22.2 | ) | 0.8 | > 0.9999 |
|  | Antidiabetics |  |  | 23 | ( | 19.7 | ) |  | 4 | ( | 11.4 | ) | 0.5 | 0.3219 |  | 0 | ( | 0.0 | ) | 0.0 | > 0.9999 |  | 2 | ( | 22.2 | ) | 1.2 | > 0.9999 |
|  |  | α-glucosidase inhibitors | | 18 | ( | 15.4 | ) |  | 3 | ( | 8.6 | ) | 0.5 | 0.4085 |  | 0 | ( | 0.0 | ) | 0.0 | > 0.9999 |  | 2 | ( | 22.2 | ) | 1.6 | 0.6337 |
|  |  | Sulfonylurea |  | 5 | ( | 4.3 | ) |  | 1 | ( | 2.9 | ) | 0.7 | > 0.9999 |  | 0 | ( | 0.0 | ) | 0.0 | > 0.9999 |  | 0 | ( | 0.0 | ) | 0.0 | > 0.9999 |
|  |  | Glinide |  | 3 | ( | 2.6 | ) |  | 0 | ( | 0.0 | ) | 0.0 | > 0.9999 |  | 0 | ( | 0.0 | ) | 0.0 | > 0.9999 |  | 0 | ( | 0.0 | ) | 0.0 | > 0.9999 |
|  |  | Insulin |  | 2 | ( | 1.7 | ) |  | 1 | ( | 2.9 | ) | 1.7 | 0.5467 |  | 0 | ( | 0.0 | ) | 0.0 | > 0.9999 |  | 0 | ( | 0.0 | ) | 0.0 | > 0.9999 |
|  |  | Biguanide |  | 1 | ( | 0.9 | ) |  | 0 | ( | 0.0 | ) | 0.0 | > 0.9999 |  | 0 | ( | 0.0 | ) | 0.0 | > 0.9999 |  | 0 | ( | 0.0 | ) | 0.0 | > 0.9999 |
|  |  | Dipeptidyl peptidase 4 inhibitor | | 1 | ( | 0.9 | ) |  | 0 | ( | 0.0 | ) | 0.0 | > 0.9999 |  | 0 | ( | 0.0 | ) | 0.0 | > 0.9999 |  | 0 | ( | 0.0 | ) | 0.0 | > 0.9999 |
|  | Immunosuppressants | |  | 10 | ( | 8.5 | ) |  | 5 | ( | 14.3 | ) | 1.8 | 0.3380 |  | 0 | ( | 0.0 | ) | 0.0 | > 0.9999 |  | 1 | ( | 11.1 | ) | 1.3 | 0.5729 |
|  | Anti-cancer agents | |  | 4 | ( | 3.4 | ) |  | 2 | ( | 5.7 | ) | 1.7 | 0.6217 |  | 0 | ( | 0.0 | ) | 0.0 | > 0.9999 |  | 3 | ( | 33.3 | ) | 14.1 | 0.0078 |
|  | Antihypertensives | |  | 15 | ( | 12.8 | ) |  | 2 | ( | 5.7 | ) | 0.4 | 0.3623 |  | 1 | ( | 33.3 | ) | 3.4 | 0.4051 |  | 1 | ( | 11.1 | ) | 0.9 | > 0.9999 |
|  |  | Calcium antagonist |  | 9 | ( | 7.7 | ) |  | 1 | ( | 2.9 | ) | 0.4 | 0.4553 |  | 0 | ( | 0.0 | ) | 0.0 | > 0.9999 |  | 0 | ( | 0.0 | ) | 0.0 | > 0.9999 |
|  |  | β-blocker |  | 5 | ( | 4.3 | ) |  | 1 | ( | 2.9 | ) | 0.7 | > 0.9999 |  | 1 | ( | 33.3 | ) | 11.2 | 0.1507 |  | 1 | ( | 11.1 | ) | 2.8 | 0.3650 |
|  |  | Angiotensin II receptor blocker | | 7 | ( | 6.0 | ) |  | 1 | ( | 2.9 | ) | 0.5 | 0.6824 |  | 1 | ( | 33.3 | ) | 7.9 | 0.2367 |  | 0 | ( | 0.0 | ) | 0.0 | > 0.9999 |
|  |  | Angiotensin converting enzyme inhibitor | | 0 | ( | 0.0 | ) |  | 1 | ( | 2.9 | ) | n.d | 0.2303 |  | 0 | ( | 0.0 | ) | n.d | > 0.9999 |  | 0 | ( | 0.0 | ) | n.d | > 0.9999 |
|  |  | α-blocker |  | 1 | ( | 0.9 | ) |  | 0 | ( | 0.0 | ) | 0.0 | > 0.9999 |  | 0 | ( | 0.0 | ) | 0.0 | > 0.9999 |  | 0 | ( | 0.0 | ) | 0.0 | > 0.9999 |
|  | Diuretics |  |  | 1 | ( | 0.9 | ) |  | 2 | ( | 5.7 | ) | 7.0 | 0.1327 |  | 1 | ( | 33.3 | ) | 58.0 | 0.0961 |  | 1 | ( | 11.1 | ) | 14.5 | 0.1383 |
|  | Digitalis |  |  | 3 | ( | 2.6 | ) |  | 0 | ( | 0.0 | ) | 0.0 | > 0.9999 |  | 0 | ( | 0.0 | ) | 0.0 | > 0.9999 |  | 1 | ( | 11.1 | ) | 4.8 | 0.2593 |
|  | Antiarrythmics |  |  | 1 | ( | 0.9 | ) |  | 0 | ( | 0.0 | ) | 0.0 | > 0.9999 |  | 0 | ( | 0.0 | ) | 0.0 | > 0.9999 |  | 1 | ( | 11.1 | ) | 14.5 | 0.1383 |
|  | Antithrombotics | |  | 10 | ( | 8.5 | ) |  | 0 | ( | 0.0 | ) | 0.0 | 0.1175 |  | 0 | ( | 0.0 | ) | 0.0 | > 0.9999 |  | 2 | ( | 22.2 | ) | 3.1 | 0.2053 |
|  |  | Anticoagulants |  | 4 | ( | 3.4 | ) |  | 0 | ( | 0.0 | ) | 0.0 | 0.5740 |  | 0 | ( | 0.0 | ) | 0.0 | > 0.9999 |  | 0 | ( | 0.0 | ) | 0.0 | > 0.9999 |
|  |  | Antiplatelets |  | 7 | ( | 6.0 | ) |  | 0 | ( | 0.0 | ) | 0.0 | 0.3530 |  | 0 | ( | 0.0 | ) | 0.0 | > 0.9999 |  | 2 | ( | 22.2 | ) | 4.5 | 0.1257 |
|  | Bronchodilators | |  | 2 | ( | 1.7 | ) |  | 1 | ( | 2.9 | ) | 1.7 | 0.5467 |  | 0 | ( | 0.0 | ) | 0.0 | > 0.9999 |  | 0 | ( | 0.0 | ) | 0.0 | > 0.9999 |
|  | Gastric acid secretion inhibitors | |  | 13 | ( | 11.1 | ) |  | 6 | ( | 17.1 | ) | 1.7 | 0.3842 |  | 0 | ( | 0.0 | ) | 0.0 | > 0.9999 |  | 1 | ( | 11.1 | ) | 1.0 | > 0.9999 |
|  |  | Proton pump inhibitors | | 8 | ( | 6.8 | ) |  | 6 | ( | 17.1 | ) | 2.8 | 0.0644 |  | 0 | ( | 0.0 | ) | 0.0 | > 0.9999 |  | 1 | ( | 11.1 | ) | 1.7 | 0.4984 |
|  |  | Histamine-2 receptor antagonists | | 5 | ( | 4.3 | ) |  | 1 | ( | 2.9 | ) | 0.7 | > 0.9999 |  | 0 | ( | 0.0 | ) | 0.0 | > 0.9999 |  | 0 | ( | 0.0 | ) | 0.0 | > 0.9999 |
|  | 5-aminosalicylates or salicylazosulfapyridine | | | 15 | ( | 12.8 | ) |  | 0 | ( | 0.0 | ) | 0.0 | 0.0229 |  | 0 | ( | 0.0 | ) | 0.0 | > 0.9999 |  | 0 | ( | 0.0 | ) | 0.0 | 0.5978 |
|  | Nonsteroidal antiinflammatory drugs | | | 2 | ( | 1.7 | ) |  | 1 | ( | 2.9 | ) | 1.7 | 0.5467 |  | 0 | ( | 0.0 | ) | 0.0 | > 0.9999 |  | 0 | ( | 0.0 | ) | 0.0 | > 0.9999 |
|  | Antibiotics |  |  | 6 | ( | 5.1 | ) |  | 3 | ( | 8.6 | ) | 1.7 | 0.4307 |  | 0 | ( | 0.0 | ) | 0.0 | > 0.9999 |  | 0 | ( | 0.0 | ) | 0.0 | > 0.9999 |
|  |  | Trimethoprim-sulfamethoxazole | | 3 | ( | 2.6 | ) |  | 3 | ( | 8.6 | ) | 3.6 | 0.1353 |  | 0 | ( | 0.0 | ) | 0.0 | > 0.9999 |  | 0 | ( | 0.0 | ) | 0.0 | > 0.9999 |
|  | Laxatives |  |  | 6 | ( | 5.1 | ) |  | 2 | ( | 5.7 | ) | 1.1 | > 0.9999 |  | 0 | ( | 0.0 | ) | 0.0 | > 0.9999 |  | 2 | ( | 22.2 | ) | 5.3 | 0.1016 |
|  | Bisphophonates | |  | 2 | ( | 1.7 | ) |  | 0 | ( | 0.0 | ) | 0.0 | > 0.9999 |  | 0 | ( | 0.0 | ) | 0.0 | > 0.9999 |  | 0 | ( | 0.0 | ) | 0.0 | > 0.9999 |
|  | Statins / ezetimib/ fibrates | |  | 12 | ( | 10.3 | ) |  | 0 | ( | 0.0 | ) | 0.0 | 0.0691 |  | 1 | ( | 33.3 | ) | 4.4 | 0.3167 |  | 1 | ( | 11.1 | ) | 1.1 | > 0.9999 |
|  | Hypnotics |  |  | 2 | ( | 1.7 | ) |  | 0 | ( | 0.0 | ) | 0.0 | > 0.9999 |  | 0 | ( | 0.0 | ) | 0.0 | > 0.9999 |  | 0 | ( | 0.0 | ) | 0.0 | > 0.9999 |
|  | Psychotropics |  |  | 4 | ( | 3.4 | ) |  | 1 | ( | 2.9 | ) | 0.8 | > 0.9999 |  | 0 | ( | 0.0 | ) | 0.0 | > 0.9999 |  | 1 | ( | 11.1 | ) | 3.5 | 0.3139 |
|  | Prostatic hypertrophy drugs | |  | 2 | ( | 1.7 | ) |  | 1 | ( | 2.9 | ) | 1.7 | 0.5467 |  | 0 | ( | 0.0 | ) | 0.0 | > 0.9999 |  | 1 | ( | 11.1 | ) | 7.2 | 0.2008 |
|  | Allopurinol / benzbromaron | |  | 2 | ( | 1.7 | ) |  | 0 | ( | 0.0 | ) | 0.0 | > 0.9999 |  | 1 | ( | 33.3 | ) | 28.8 | 0.1327 |  | 1 | ( | 11.1 | ) | 7.2 | 0.2008 |
|  | Levothyroxine |  |  | 2 | ( | 1.7 | ) |  | 1 | ( | 2.9 | ) | 1.7 | 0.5467 |  | 1 | ( | 33.3 | ) | 28.8 | 0.0774 |  | 0 | ( | 0.0 | ) | 0.0 | > 0.9999 |
|  | Herbal medicine | |  | 5 | ( | 4.3 | ) |  | 1 | ( | 2.9 | ) | 0.7 | > 0.9999 |  | 0 | ( | 0.0 | ) | 0.0 | > 0.9999 |  | 1 | ( | 11.1 | ) | 2.8 | 0.3650 |
| Comobidities and/or past medical history | | | | 98 | ( | 83.8 | ) |  | 28 | ( | 80.0 | ) | 0.8 | 0.6042 |  | 3 | ( | 37.5 | ) | n.d | 0.3843 |  | 11 | ( | 91.7 | ) | 2.1 | 0.6903 |
|  | Gastroduodenal diseases | |  | 34 | ( | 29.1 | ) |  | 10 | ( | 28.6 | ) | 1.0 | 0.9554 |  | 0 | ( | 0.0 | ) | 0.0 | > 0.9999 |  | 1 | ( | 8.3 | ) | 0.2 | 0.1784 |
|  |  | Inflammatory bowel disease | | 13 | ( | 11.1 | ) |  | 2 | ( | 5.7 | ) | 0.5 | 0.5222 |  | 0 | ( | 0.0 | ) | 0.0 | > 0.9999 |  | 1 | ( | 8.3 | ) | 0.7 | > 0.9999 |
|  |  |  | Ulcerative colitis | 12 | ( | 10.3 | ) |  | 0 | ( | 0.0 | ) | 0.0 | 0.0691 |  | 0 | ( | 0.0 | ) | 0.0 | > 0.9999 |  | 1 | ( | 8.3 | ) | 0.8 | > 0.9999 |
|  |  |  | Crohn's disease | 1 | ( | 0.9 | ) |  | 1 | ( | 2.9 | ) | 3.4 | 0.4087 |  | 0 | ( | 0.0 | ) | 0.0 | > 0.9999 |  | 0 | ( | 0.0 | ) | 0.0 | > 0.9999 |
|  |  |  | Behcet's disease | 0 | ( | 0.0 | ) |  | 1 | ( | 2.9 | ) | n.d | 0.2303 |  | 0 | ( | 0.0 | ) | n.d | > 0.9999 |  | 0 | ( | 0.0 | ) | n.d | > 0.9999 |
|  |  | Carcinoma |  | 11 | ( | 9.4 | ) |  | 2 | ( | 5.7 | ) | 0.6 | 0.7334 |  | 0 | ( | 0.0 | ) | 0.0 | > 0.9999 |  | 0 | ( | 0.0 | ) | 0.0 | 0.5977 |
|  |  |  | Esophegeal carcinoma | 0 | ( | 0.0 | ) |  | 1 | ( | 2.9 | ) | n.d | 0.2303 |  | 0 | ( | 0.0 | ) | n.d | > 0.9999 |  | 0 | ( | 0.0 | ) | n.d | > 0.9999 |
|  |  |  | Gastric carcinoma | 3 | ( | 2.6 | ) |  | 0 | ( | 0.0 | ) | 0.0 | > 0.9999 |  | 0 | ( | 0.0 | ) | 0.0 | > 0.9999 |  | 0 | ( | 0.0 | ) | 0.0 | > 0.9999 |
|  |  |  | Colorectal carcinoma | 8 | ( | 6.8 | ) |  | 1 | ( | 2.9 | ) | 0.4 | 0.6853 |  | 0 | ( | 0.0 | ) | 0.0 | > 0.9999 |  | 1 | ( | 8.3 | ) | 1.2 | 0.5970 |
|  |  | Colorectal polyp |  | 5 | ( | 4.3 | ) |  | 1 | ( | 2.9 | ) | 0.7 | > 0.9999 |  | 0 | ( | 0.0 | ) | 0.0 | > 0.9999 |  | 0 | ( | 0.0 | ) | 0.0 | > 0.9999 |
|  |  | Bowel obstruction |  | 1 | ( | 0.9 | ) |  | 3 | ( | 8.6 | ) | 10.9 | 0.0383 |  | 0 | ( | 0.0 | ) | 0.0 | > 0.9999 |  | 0 | ( | 0.0 | ) | 0.0 | > 0.9999 |
|  |  | Others |  | 2 | ( | 1.7 | ) |  | 3 | ( | 8.6 | ) | 5.4 | 0.0804 |  | 0 | ( | 0.0 | ) | 0.0 | > 0.9999 |  | 0 | ( | 0.0 | ) | 0.0 | > 0.9999 |
|  |  |  | Esophegeal candidiasis | 0 | ( | 0.0 | ) |  | 1 | ( | 2.9 | ) | n.d | 0.2303 |  | 0 | ( | 0.0 | ) | n.d | > 0.9999 |  | 0 | ( | 0.0 | ) | n.d | > 0.9999 |
|  |  |  | Gastroesophageal reflux disease | 1 | ( | 0.9 | ) |  | 0 | ( | 0.0 | ) | 0.0 | > 0.9999 |  | 0 | ( | 0.0 | ) | 0.0 | > 0.9999 |  | 0 | ( | 0.0 | ) | 0.0 | > 0.9999 |
|  |  |  | Peptic ulcer disease | 1 | ( | 0.9 | ) |  | 1 | ( | 2.9 | ) | 3.4 | 0.4087 |  | 0 | ( | 0.0 | ) | 0.0 | > 0.9999 |  | 0 | ( | 0.0 | ) | 0.0 | > 0.9999 |
|  |  |  | Ischemic colitis | 0 | ( | 0.0 | ) |  | 1 | ( | 2.9 | ) | n.d | 0.2303 |  | 0 | ( | 0.0 | ) | n.d | > 0.9999 |  | 0 | ( | 0.0 | ) | n.d | > 0.9999 |
|  | Hepatobiliarypancreatic disease | |  | 8 | ( | 6.8 | ) |  | 1 | ( | 2.9 | ) | 0.4 | 0.6853 |  | 0 | ( | 0.0 | ) | 0.0 | > 0.9999 |  | 1 | ( | 8.3 | ) | 1.2 | 0.5970 |
|  |  | Hepatic hemangioma | | 1 | ( | 0.9 | ) |  | 0 | ( | 0.0 | ) | 0.0 | > 0.9999 |  | 0 | ( | 0.0 | ) | 0.0 | > 0.9999 |  | 0 | ( | 0.0 | ) | 0.0 | > 0.9999 |
|  |  | Chronic hepatitis |  | 2 | ( | 1.7 | ) |  | 0 | ( | 0.0 | ) | 0.0 | > 0.9999 |  | 0 | ( | 0.0 | ) | 0.0 | > 0.9999 |  | 0 | ( | 0.0 | ) | 0.0 | > 0.9999 |
|  |  | Cirrhosis |  | 1 | ( | 0.9 | ) |  | 0 | ( | 0.0 | ) | 0.0 | > 0.9999 |  | 0 | ( | 0.0 | ) | 0.0 | > 0.9999 |  | 0 | ( | 0.0 | ) | 0.0 | > 0.9999 |
|  |  | Hepatic carcinoma |  | 0 | ( | 0.0 | ) |  | 0 | ( | 0.0 | ) | n.d | > 0.9999 |  | 0 | ( | 0.0 | ) | n.d | > 0.9999 |  | 1 | ( | 8.3 | ) | n.d | > 0.9999 |
|  |  | Cholecystitis |  | 3 | ( | 2.6 | ) |  | 1 | ( | 2.9 | ) | 1.1 | > 0.9999 |  | 0 | ( | 0.0 | ) | 0.0 | > 0.9999 |  | 0 | ( | 0.0 | ) | 0.0 | > 0.9999 |
|  |  | Chroinc pancreatitis | | 1 | ( | 0.9 | ) |  | 0 | ( | 0.0 | ) | 0.0 | > 0.9999 |  | 0 | ( | 0.0 | ) | 0.0 | > 0.9999 |  | 0 | ( | 0.0 | ) | 0.0 | > 0.9999 |
|  | Diabetes mellitus | |  | 21 | ( | 17.9 | ) |  | 5 | ( | 14.3 | ) | 0.8 | 0.7991 |  | 0 | ( | 0.0 | ) | 0.0 | 0.3591 |  | 3 | ( | 25.0 | ) | 1.5 | 0.6956 |
|  | Chronic lung disease | |  | 19 | ( | 16.2 | ) |  | 7 | ( | 20.0 | ) | 1.3 | 0.6042 |  | 1 | ( | 12.5 | ) | 2.6 | > 0.9999 |  | 1 | ( | 8.3 | ) | 0.5 | 0.6903 |
|  | Autoimmune disease | |  | 18 | ( | 15.4 | ) |  | 11 | ( | 31.4 | ) | 2.5 | 0.0341 |  | 0 | ( | 0.0 | ) | 0.0 | 0.6027 |  | 1 | ( | 8.3 | ) | 0.5 | > 0.9999 |
|  | Hypertension |  |  | 10 | ( | 8.5 | ) |  | 1 | ( | 2.9 | ) | 0.3 | 0.4582 |  | 1 | ( | 12.5 | ) | 5.4 | 0.1383 |  | 1 | ( | 8.3 | ) | 1.0 | > 0.9999 |
|  | Heart disease |  |  | 9 | ( | 7.7 | ) |  | 1 | ( | 2.9 | ) | 0.4 | 0.4553 |  | 0 | ( | 0.0 | ) | 0.0 | 0.4706 |  | 1 | ( | 8.3 | ) | 1.1 | > 0.9999 |
|  | Dyslipidemia |  |  | 7 | ( | 6.0 | ) |  | 0 | ( | 0.0 | ) | 0.0 | 0.3530 |  | 0 | ( | 0.0 | ) | 0.0 | 0.3823 |  | 1 | ( | 8.3 | ) | 1.4 | 0.5526 |
|  | Hematological disease | |  | 6 | ( | 5.1 | ) |  | 1 | ( | 2.9 | ) | 0.5 | > 0.9999 |  | 0 | ( | 0.0 | ) | 0.0 | > 0.9999 |  | 0 | ( | 0.0 | ) | 0.0 | > 0.9999 |
|  |  | Bone marrow transplantation | | 2 | ( | 1.7 | ) |  | 1 | ( | 2.9 | ) | 1.7 | 0.5467 |  | 0 | ( | 0.0 | ) | 0.0 | > 0.9999 |  | 0 | ( | 0.0 | ) | 0.0 | > 0.9999 |
|  | Kideny disease | |  | 2 | ( | 1.7 | ) |  | 2 | ( | 5.7 | ) | 3.5 | 0.2272 |  | 0 | ( | 0.0 | ) | 0.0 | > 0.9999 |  | 0 | ( | 0.0 | ) | 0.0 | > 0.9999 |
|  | Hyperuricemia |  |  | 2 | ( | 1.7 | ) |  | 0 | ( | 0.0 | ) | 0.0 | > 0.9999 |  | 0 | ( | 0.0 | ) | 0.0 | > 0.9999 |  | 1 | ( | 8.3 | ) | 5.2 | 0.2557 |
|  | Psychiatric diseases | |  | 3 | ( | 2.6 | ) |  | 0 | ( | 0.0 | ) | 0.0 | > 0.9999 |  | 0 | ( | 0.0 | ) | 0.0 | > 0.9999 |  | 1 | ( | 8.3 | ) | 3.5 | 0.3266 |
|  | Neurological diseases | |  | 3 | ( | 2.6 | ) |  | 1 | ( | 2.9 | ) | 1.1 | > 0.9999 |  | 0 | ( | 0.0 | ) | 0.0 | > 0.9999 |  | 0 | ( | 0.0 | ) | 0.0 | > 0.9999 |
|  | Peripheral vascular disease | |  | 0 | ( | 0.0 | ) |  | 1 | ( | 2.9 | ) | n.d | 0.2303 |  | 0 | ( | 0.0 | ) | n.d | > 0.9999 |  | 0 | ( | 0.0 | ) | n.d | > 0.9999 |
|  | Endocine disease | |  | 2 | ( | 1.7 | ) |  | 1 | ( | 2.9 | ) | 1.7 | 0.5467 |  | 1 | ( | 12.5 | ) | 28.8 | 0.1351 |  | 0 | ( | 0.0 | ) | 0.0 | > 0.9999 |
|  | Cancer except the digestive or hematologic system | | | 4 | ( | 3.4 | ) |  | 3 | ( | 8.6 | ) | 2.6 | 0.1996 |  | 0 | ( | 0.0 | ) | 0.0 | > 0.9999 |  | 2 | ( | 16.7 | ) | 5.7 | 0.0967 |
| Segments involved | |  |  |  |  |  |  |  |  |  |  |  |  |  |  |  |  |  |  |  |  |  |  |  |  |  |  |  |
|  | Large bowel only | |  | 92 | ( | 78.6 | ) |  | 21 | ( | 60.0 | ) | 0.4 | 0.0268 |  | 3 | ( | 37.5 | ) | n.d | 0.6030 |  | 3 | ( | 25.0 | ) | 0.1 | 0.0009 |
|  |  | Right-sided colon only | | 70 | ( | 59.8 | ) |  | 10 | ( | 28.6 | ) | 0.3 | 0.0012 |  | 2 | ( | 25.0 | ) | 1.3 | 0.4425 |  | 2 | ( | 16.7 | ) | 0.1 | 0.0103 |
|  |  | Left-sided colon only | | 17 | ( | 14.5 | ) |  | 6 | ( | 17.1 | ) | 1.2 | 0.7051 |  | 1 | ( | 12.5 | ) | 2.9 | > 0.9999 |  | 1 | ( | 8.3 | ) | 0.6 | > 0.9999 |
|  |  | Rectum only |  | 1 | ( | 0.9 | ) |  | 0 | ( | 0.0 | ) | 0.0 | > 0.9999 |  | 0 | ( | 0.0 | ) | 0.0 | > 0.9999 |  | 0 | ( | 0.0 | ) | 0.0 | > 0.9999 |
|  |  | Righ- and left-sided colon | | 1 | ( | 0.9 | ) |  | 5 | ( | 14.3 | ) | 19.3 | 0.0026 |  | 0 | ( | 0.0 | ) | 0.0 | > 0.9999 |  | 0 | ( | 0.0 | ) | 0.0 | > 0.9999 |
|  |  | Left-sided colon and rectum | | 1 | ( | 0.9 | ) |  | 0 | ( | 0.0 | ) | 0.0 | > 0.9999 |  | 0 | ( | 0.0 | ) | 0.0 | > 0.9999 |  | 0 | ( | 0.0 | ) | 0.0 | > 0.9999 |
|  |  | Throughout the large bowel | | 2 | ( | 1.7 | ) |  | 0 | ( | 0.0 | ) | 0.0 | > 0.9999 |  | 0 | ( | 0.0 | ) | 0.0 | 0.0991 |  | 0 | ( | 0.0 | ) | 0.0 | > 0.9999 |
|  | Small bowel only | |  | 18 | ( | 15.4 | ) |  | 9 | ( | 25.7 | ) | 1.9 | 0.1607 |  | 0 | ( | 0.0 | ) | 0.0 | > 0.9999 |  | 6 | ( | 50.0 | ) | 6.6 | 0.0058 |
|  |  | Ileum only |  | 8 | ( | 6.8 | ) |  | 2 | ( | 5.7 | ) | 0.8 | > 0.9999 |  | 0 | ( | 0.0 | ) | 0.0 | > 0.9999 |  | 5 | ( | 41.7 | ) | 11.4 | 0.0016 |
|  |  | Jejunum only |  | 7 | ( | 6.0 | ) |  | 3 | ( | 8.6 | ) | 1.5 | 0.6974 |  | 0 | ( | 0.0 | ) | 0.0 | > 0.9999 |  | 1 | ( | 8.3 | ) | 1.6 | 0.5230 |
|  |  | Ileum and jejunum |  | 3 | ( | 2.6 | ) |  | 4 | ( | 11.4 | ) | 4.9 | 0.0496 |  | 0 | ( | 0.0 | ) | 0.0 | > 0.9999 |  | 0 | ( | 0.0 | ) | 0.0 | > 0.9999 |
|  | Combined |  |  | 7 | ( | 6.0 | ) |  | 4 | ( | 11.4 | ) | 2.0 | 0.2778 |  | 0 | ( | 0.0 | ) | 0.0 | > 0.9999 |  | 2 | ( | 16.7 | ) | 3.5 | 0.1732 |
|  |  | Ileum and right-sided colon | | 1 | ( | 0.9 | ) |  | 2 | ( | 5.7 | ) | 7.0 | 0.1327 |  | 0 | ( | 0.0 | ) | 0.0 | > 0.9999 |  | 0 | ( | 0.0 | ) | 0.0 | > 0.9999 |
|  |  | Jejunum and right-sided colon | | 1 | ( | 0.9 | ) |  | 0 | ( | 0.0 | ) | 0.0 | > 0.9999 |  | 0 | ( | 0.0 | ) | 0.0 | > 0.9999 |  | 0 | ( | 0.0 | ) | 0.0 | > 0.9999 |
|  |  | Ileum, right- and left-sided colon | | 2 | ( | 1.7 | ) |  | 0 | ( | 0.0 | ) | 0.0 | > 0.9999 |  | 0 | ( | 0.0 | ) | 0.0 | > 0.9999 |  | 1 | ( | 8.3 | ) | 5.8 | 0.2380 |
|  |  | Jejunum, ileum, right- and left-sided colon | | 1 | ( | 0.9 | ) |  | 2 | ( | 5.7 | ) | 7.0 | 0.1327 |  | 0 | ( | 0.0 | ) | 0.0 | > 0.9999 |  | 0 | ( | 0.0 | ) | 0.0 | > 0.9999 |
|  |  | Esophagus, stomach, small bowel | | 1 | ( | 0.9 | ) |  | 0 | ( | 0.0 | ) | 0.0 | > 0.9999 |  | 0 | ( | 0.0 | ) | 0.0 | > 0.9999 |  | 1 | ( | 8.3 | ) | 11.6 | 0.1651 |
|  |  | Esophagus, stomach, small bowel, and colon | | 1 | ( | 0.9 | ) |  | 0 | ( | 0.0 | ) | 0.0 | > 0.9999 |  | 0 | ( | 0.0 | ) | 0.0 | > 0.9999 |  | 0 | ( | 0.0 | ) | 0.0 | > 0.9999 |
| Complicating pnuematosis intestinalis | | |  | 11 | ( | 9.4 | ) |  | 7 | ( | 20.0 | ) | 2.4 | 0.0887 |  | 0 | ( | 0.0 | ) | 0.0 | > 0.9999 |  | 8 | ( | 66.7 | ) | 25.7 | < 0.0001 |
| Outcome of pneumatosi intestinalis | | |  |  |  |  |  |  |  |  |  |  |  |  |  |  |  |  |  |  |  |  |  |  |  |  |  |  |
|  |  | Improvement |  | 79 | ( | 67.5 | ) |  | 28 | ( | 80.0 | ) | 1.9 | 0.1560 |  | 2 | ( | 28.6 | ) | 1.0 | 0.4322 |  | 10 | ( | 83.3 | ) | 2.4 | 0.3399 |
|  |  | No change |  | 33 | ( | 28.2 | ) |  | 6 | ( | 17.1 | ) | 0.5 | 0.1886 |  | 0 | ( | 0.0 | ) | 0.0 | 0.1933 |  | 1 | ( | 8.3 | ) | 0.2 | 0.1815 |
|  |  | Exacerbation |  | 5 | ( | 4.3 | ) |  | 1 | ( | 2.9 | ) | 0.7 | > 0.9999 |  | 1 | ( | 14.3 | ) | 11.2 | 0.2272 |  | 1 | ( | 8.3 | ) | 2.0 | 0.4502 |
|  |  | Recurrence |  | 0 | ( | 0.0 | ) |  | 1 | ( | 2.9 | ) | n.d | 0.2303 |  | 0 | ( | 0.0 | ) | n.d | > 0.9999 |  | 0 | ( | 0.0 | ) | n.d | > 0.9999 |
|  |  | Death |  | 6 | ( | 5.1 | ) |  | 1 | ( | 2.9 | ) | 0.5 | > 0.9999 |  | 0 | ( | 0.0 | ) | n.d | > 0.9999 |  | 1 | ( | 8.3 | ) | 1.7 | 0.5038 |
