# Supplementary material for: Multicenter epidemiological survey of pneumatosis intestinalis in Japan
Source: BMC Gastroenterol. 2022 May 31;22:272. doi: 10.1186/s12876-022-02343-5 (PMC9153137; doi:10.1186/s12876-022-02343-5)
Supplement: Supplementary file 5 — Additional file 5. Supplementary Table 5. [file 12876_2022_2343_MOESM5_ESM.docx]

| Supplementary Table 5. Outcomes of pneumatosis intestinalis | | | | | | | | | | | | | | | | | | | | | | | |
| --- | --- | --- | --- | --- | --- | --- | --- | --- | --- | --- | --- | --- | --- | --- | --- | --- | --- | --- | --- | --- | --- | --- | --- |
|  |  |  |  | Improvement | | | |  | No change | | | | | | |  | Exacerbation | | | | | | |
| Characteristics | |  |  | n | ( | % | ) |  | n | ( | % | ) |  | Crude  OR | *P* |  | n | ( | % | ) |  | Crude  OR | *P* |
| No.patients | |  |  | 119 | ( | 50.6 | ) |  | 40 | ( | 13.0 | ) |  |  |  |  | 8 | ( | 2.6 | ) |  |  |  |
| Men/women | |  |  | 60/59 | ( | 1.0 | ) |  | 19/21 | ( | 0.9 | ) |  | 0.8 |  |  | 8/0 | ( | n.d | ) |  | n.d | 0.0090 |
| Median age of onset (y) | | |  | 64 (range 9-87) | | | |  | 65 (range 38-84) | | | | | | 0.8460 |  | 68 (range 55-91) | | | | | | 0.3402 |
| Exposure to organic solvents | | |  | 2 | ( | 1.7 | ) |  | 0 | ( | 0.0 | ) |  | 0.0 | > 0.9999 |  | 0 | ( | 0.0 | ) |  | 0.0 | > 0.9999 |
| Medications used | | |  | 91 | ( | 76.5 | ) |  | 26 | ( | 76.5 | ) |  | 0.6 | 0.1546 |  | 5 | ( | 83.3 | ) |  | 0.5 | 0.4027 |
|  | Corticosteroid | |  | 32 | ( | 26.9 | ) |  | 12 | ( | 35.3 | ) |  | 1.2 | 0.7038 |  | 0 | ( | 0.0 | ) |  | 0.0 | 0.2002 |
|  | Antidiabetics | |  | 25 | ( | 21.0 | ) |  | 2 | ( | 5.9 | ) |  | 0.2 | 0.0262 |  | 2 | ( | 33.3 | ) |  | 1.3 | 0.6775 |
|  |  | α-glucosidase inhibitors | | 20 | ( | 16.8 | ) |  | 1 | ( | 2.9 | ) |  | 0.1 | 0.0276 |  | 2 | ( | 33.3 | ) |  | 1.7 | 0.6264 |
|  |  | Sulfonylurea |  | 4 | ( | 3.4 | ) |  | 1 | ( | 2.9 | ) |  | 0.7 | > 0.9999 |  | 1 | ( | 16.7 | ) |  | 4.1 | 0.2816 |
|  |  | Glinide |  | 3 | ( | 2.5 | ) |  | 0 | ( | 0.0 | ) |  | 0.0 | 0.5728 |  | 0 | ( | 0.0 | ) |  | 0.0 | > 0.9999 |
|  |  | Insulin |  | 3 | ( | 2.5 | ) |  | 0 | ( | 0.0 | ) |  | 0.0 | 0.5728 |  | 0 | ( | 0.0 | ) |  | 0.0 | > 0.9999 |
|  |  | Biguanide |  | 1 | ( | 0.8 | ) |  | 0 | ( | 0.0 | ) |  | 0.0 | > 0.9999 |  | 0 | ( | 0.0 | ) |  | 0.0 | > 0.9999 |
|  |  | Dipeptidyl peptidase 4 inhibitor | | 1 | ( | 0.8 | ) |  | 0 | ( | 0.0 | ) |  | 0.0 | > 0.9999 |  | 0 | ( | 0.0 | ) |  | 0.0 | > 0.9999 |
|  | Immunosuppressants | |  | 10 | ( | 8.4 | ) |  | 5 | ( | 14.7 | ) |  | 1.6 | 0.5318 |  | 1 | ( | 16.7 | ) |  | 1.6 | 0.5260 |
|  | Anti-cancer agents | |  | 8 | ( | 6.7 | ) |  | 1 | ( | 2.9 | ) |  | 0.4 | > 0.9999 |  | 0 | ( | 0.0 | ) |  | 0.0 | > 0.9999 |
|  | Antihypertensives | |  | 10 | ( | 8.4 | ) |  | 6 | ( | 17.6 | ) |  | 1.9 | 0.2354 |  | 3 | ( | 50.0 | ) |  | 6.5 | 0.0351 |
|  |  | Calcium antagonist | | 5 | ( | 4.2 | ) |  | 4 | ( | 11.8 | ) |  | 2.5 | 0.2306 |  | 1 | ( | 16.7 | ) |  | 3.3 | 0.3287 |
|  |  | β-blocker |  | 6 | ( | 5.0 | ) |  | 3 | ( | 8.8 | ) |  | 1.5 | 0.6926 |  | 0 | ( | 0.0 | ) |  | 0.0 | > 0.9999 |
|  |  | Angiotensin II receptor blocker | | 5 | ( | 4.2 | ) |  | 1 | ( | 2.9 | ) |  | 0.6 | > 0.9999 |  | 2 | ( | 33.3 | ) |  | 7.6 | 0.0624 |
|  |  | Angiotensin converting enzyme inhibitor | | 1 | ( | 0.8 | ) |  | 0 | ( | 0.0 | ) |  | 0.0 | > 0.9999 |  | 0 | ( | 0.0 | ) |  | 0.0 | > 0.9999 |
|  |  | α-blocker |  | 0 | ( | 0.0 | ) |  | 0 | ( | 0.0 | ) |  | n.d | > 0.9999 |  | 1 | ( | 16.7 | ) |  | n.d | 0.0630 |
|  | Diuretics |  |  | 4 | ( | 3.4 | ) |  | 0 | ( | 0.0 | ) |  | 0.0 | 0.5728 |  | 1 | ( | 16.7 | ) |  | 4.1 | 0.2816 |
|  | Digitalis |  |  | 3 | ( | 2.5 | ) |  | 1 | ( | 2.9 | ) |  | 1.0 | > 0.9999 |  | 0 | ( | 0.0 | ) |  | 0.0 | > 0.9999 |
|  | Antiarrythmics | |  | 1 | ( | 0.8 | ) |  | 1 | ( | 2.9 | ) |  | 3.0 | 0.4410 |  | 0 | ( | 0.0 | ) |  | 0.0 | > 0.9999 |
|  | Antithrombotics | |  | 10 | ( | 8.4 | ) |  | 2 | ( | 5.9 | ) |  | 0.6 | 0.7317 |  | 0 | ( | 0.0 | ) |  | 0.0 | > 0.9999 |
|  |  | Anticoagulants |  | 3 | ( | 2.5 | ) |  | 1 | ( | 2.9 | ) |  | 1.0 | > 0.9999 |  | 0 | ( | 0.0 | ) |  | 0.0 | > 0.9999 |
|  |  | Antiplatelets |  | 8 | ( | 6.7 | ) |  | 1 | ( | 2.9 | ) |  | 0.4 | 0.4514 |  | 0 | ( | 0.0 | ) |  | 0.0 | > 0.9999 |
|  | Bronchodilators | |  | 2 | ( | 1.7 | ) |  | 0 | ( | 0.0 | ) |  | 0.0 | > 0.9999 |  | 1 | ( | 16.7 | ) |  | 8.4 | 0.1786 |
|  | Gastric acid secretion inhibitors | | | 14 | ( | 11.8 | ) |  | 6 | ( | 17.6 | ) |  | 1.3 | 0.5935 |  | 0 | ( | 0.0 | ) |  | 0.0 | 0.5966 |
|  |  | Proton pump inhibitors | | 11 | ( | 9.2 | ) |  | 4 | ( | 11.8 | ) |  | 1.1 | > 0.9999 |  | 0 | ( | 0.0 | ) |  | 0.0 | > 0.9999 |
|  |  | Histamine-2 receptor antagonists | | 4 | ( | 3.4 | ) |  | 2 | ( | 5.9 | ) |  | 1.5 | 0.6419 |  | 0 | ( | 0.0 | ) |  | 0.0 | > 0.9999 |
|  | 5-aminosalicylates or salicylazosulfapyridine | | | 8 | ( | 6.7 | ) |  | 7 | ( | 20.6 | ) |  | 2.9 | 0.0437 |  | 0 | ( | 0.0 | ) |  | 0.0 | > 0.9999 |
|  | Nonsteroidal antiinflammatory drugs | | | 3 | ( | 2.5 | ) |  | 0 | ( | 0.0 | ) |  | 0.0 | 0.5728 |  | 0 | ( | 0.0 | ) |  | 0.0 | > 0.9999 |
|  | Antibiotics | |  | 7 | ( | 5.9 | ) |  | 2 | ( | 5.9 | ) |  | 0.8 | > 0.9999 |  | 0 | ( | 0.0 | ) |  | 0.0 | > 0.9999 |
|  |  | Trimethoprim-sulfamethoxazole | | 5 | ( | 4.2 | ) |  | 1 | ( | 2.9 | ) |  | 0.6 | > 0.9999 |  | 0 | ( | 0.0 | ) |  | 0.0 | > 0.9999 |
|  | Laxatives | |  | 8 | ( | 6.7 | ) |  | 0 | ( | 0.0 | ) |  | 0.0 | 0.2030 |  | 2 | ( | 33.3 | ) |  | 4.6 | 0.1212 |
|  | Bisphophonates | |  | 0 | ( | 0.0 | ) |  | 2 | ( | 5.9 | ) |  | n.d | 0.0621 |  | 0 | ( | 0.0 | ) |  | n.d | > 0.9999 |
|  | Statins / ezetimib/ fibrates | |  | 10 | ( | 8.4 | ) |  | 4 | ( | 11.8 | ) |  | 1.2 | 0.7520 |  | 0 | ( | 0.0 | ) |  | 0.0 | > 0.9999 |
|  | Hypnotics | |  | 2 | ( | 1.7 | ) |  | 0 | ( | 0.0 | ) |  | 0.0 | > 0.9999 |  | 0 | ( | 0.0 | ) |  | 0.0 | > 0.9999 |
|  | Psychotropics | |  | 3 | ( | 2.5 | ) |  | 2 | ( | 5.9 | ) |  | 2.0 | 0.6003 |  | 1 | ( | 16.7 | ) |  | 5.5 | 0.2316 |
|  | Prostatic hypertrophy drugs | |  | 3 | ( | 2.5 | ) |  | 0 | ( | 0.0 | ) |  | 0.0 | 0.5728 |  | 1 | ( | 16.7 | ) |  | 5.5 | 0.2316 |
|  | Allopurinol / benzbromaron | |  | 3 | ( | 2.5 | ) |  | 0 | ( | 0.0 | ) |  | 0.0 | 0.5728 |  | 0 | ( | 0.0 | ) |  | 0.0 | > 0.9999 |
|  | Levothyroxine | |  | 3 | ( | 2.5 | ) |  | 1 | ( | 2.9 | ) |  | 1.0 | > 0.9999 |  | 0 | ( | 0.0 | ) |  | 0.0 | > 0.9999 |
|  | Herbal medicine | |  | 3 | ( | 2.5 | ) |  | 3 | ( | 8.8 | ) |  | 3.1 | > 0.9999 |  | 1 | ( | 16.7 | ) |  | 5.5 | 0.2316 |
| Comobidities and/or past medical history | | | | 98 | ( | 83.1 | ) |  | 35 | ( | 67.3 | ) |  | 1.5 | 0.6209 |  | 7 | ( | 87.5 | ) |  | 1.5 | > 0.9999 |
|  | Gastrointestinal diseases | |  | 33 | ( | 28.0 | ) |  | 12 | ( | 23.1 | ) |  | 1.1 | 0.8054 |  | 0 | ( | 0.0 | ) |  | 0.0 | 0.1097 |
|  |  | Inflammatory bowel disease | | 10 | ( | 8.5 | ) |  | 6 | ( | 11.5 | ) |  | 1.9 | 0.2371 |  | 0 | ( | 0.0 | ) |  | 0.0 | > 0.9999 |
|  |  |  | Ulcerative colitis | 7 | ( | 5.9 | ) |  | 6 | ( | 11.5 | ) |  | 2.8 | 0.0713 |  | 0 | ( | 0.0 | ) |  | 0.0 | > 0.9999 |
|  |  |  | Crohn's disease | 2 | ( | 1.7 | ) |  | 0 | ( | 0.0 | ) |  | 0.0 | > 0.9999 |  | 0 | ( | 0.0 | ) |  | 0.0 | > 0.9999 |
|  |  |  | Behcet's disease | 1 | ( | 0.8 | ) |  | 0 | ( | 0.0 | ) |  | 0.0 | > 0.9999 |  | 0 | ( | 0.0 | ) |  | 0.0 | > 0.9999 |
|  |  | Carcinoma |  | 9 | ( | 7.6 | ) |  | 4 | ( | 7.7 | ) |  | 1.4 | 0.7396 |  | 0 | ( | 0.0 | ) |  | 0.0 | > 0.9999 |
|  |  |  | Esophegeal carcinoma | 1 | ( | 0.8 | ) |  | 0 | ( | 0.0 | ) |  | 0.0 | > 0.9999 |  | 0 | ( | 0.0 | ) |  | 0.0 | > 0.9999 |
|  |  |  | Gastric carcinoma | 2 | ( | 1.7 | ) |  | 1 | ( | 1.9 | ) |  | 1.5 | > 0.9999 |  | 0 | ( | 0.0 | ) |  | 0.0 | > 0.9999 |
|  |  |  | Colorectal carcinoma | 6 | ( | 5.1 | ) |  | 3 | ( | 5.8 | ) |  | 1.5 | 0.6933 |  | 0 | ( | 0.0 | ) |  | 0.0 | > 0.9999 |
|  |  | Colorectal polyp |  | 5 | ( | 4.2 | ) |  | 1 | ( | 1.9 | ) |  | 0.6 | > 0.9999 |  | 0 | ( | 0.0 | ) |  | 0.0 | > 0.9999 |
|  |  | Bowel obstruction | | 3 | ( | 2.5 | ) |  | 1 | ( | 1.9 | ) |  | 1.0 | > 0.9999 |  | 0 | ( | 0.0 | ) |  | 0.0 | > 0.9999 |
|  |  | Others |  | 5 | ( | 4.2 | ) |  | 0 | ( | 0.0 | ) |  | 0.0 | 0.3308 |  | 0 | ( | 0.0 | ) |  | 0.0 | > 0.9999 |
|  |  |  | Esophegeal candidiasis | 1 | ( | 0.8 | ) |  | 0 | ( | 0.0 | ) |  | 0.0 | > 0.9999 |  | 0 | ( | 0.0 | ) |  | 0.0 | > 0.9999 |
|  |  |  | Gastroesophageal reflux disease | 1 | ( | 0.8 | ) |  | 0 | ( | 0.0 | ) |  | 0.0 | > 0.9999 |  | 0 | ( | 0.0 | ) |  | 0.0 | > 0.9999 |
|  |  |  | Peptic ulcer disease | 2 | ( | 1.7 | ) |  | 0 | ( | 0.0 | ) |  | 0.0 | > 0.9999 |  | 0 | ( | 0.0 | ) |  | 0.0 | > 0.9999 |
|  |  |  | Ischemic colitis | 1 | ( | 0.8 | ) |  | 0 | ( | 0.0 | ) |  | 0.0 | > 0.9999 |  | 0 | ( | 0.0 | ) |  | 0.0 | > 0.9999 |
|  | Hepatobiliarypancreatic disease | | | 7 | ( | 5.9 | ) |  | 1 | ( | 1.9 | ) |  | 0.4 | 0.6805 |  | 2 | ( | 25.0 | ) |  | 5.3 | 0.1016 |
|  |  | Hepatic hemangioma | | 1 | ( | 0.8 | ) |  | 0 | ( | 0.0 | ) |  | 0.0 | > 0.9999 |  | 0 | ( | 0.0 | ) |  | 0.0 | > 0.9999 |
|  |  | Chronic hepatitis |  | 2 | ( | 1.7 | ) |  | 0 | ( | 0.0 | ) |  | 0.0 | > 0.9999 |  | 0 | ( | 0.0 | ) |  | 0.0 | > 0.9999 |
|  |  | Cirrhosis |  | 0 | ( | 0.0 | ) |  | 0 | ( | 0.0 | ) |  | n.d | > 0.9999 |  | 1 | ( | 12.5 | ) |  | n.d | 0.0635 |
|  |  | Hepatic carcinoma | | 1 | ( | 0.8 | ) |  | 0 | ( | 0.0 | ) |  | 0.0 | > 0.9999 |  | 0 | ( | 0.0 | ) |  | 0.0 | 0.1800 |
|  |  | Cholecystitis |  | 2 | ( | 1.7 | ) |  | 1 | ( | 1.9 | ) |  | 1.5 | > 0.9999 |  | 1 | ( | 12.5 | ) |  | 8.4 | 0.1786 |
|  |  | Chroinc pancreatitis | | 1 | ( | 0.8 | ) |  | 0 | ( | 0.0 | ) |  | 0.0 | > 0.9999 |  | 0 | ( | 0.0 | ) |  | 0.0 | > 0.9999 |
|  | Diabetes mellitus | |  | 25 | ( | 21.2 | ) |  | 3 | ( | 5.8 | ) |  | 0.3 | 0.0568 |  | 1 | ( | 12.5 | ) |  | 0.5 | > 0.9999 |
|  | Chronic lung disease | |  | 19 | ( | 16.1 | ) |  | 7 | ( | 13.5 | ) |  | 1.1 | 0.8367 |  | 2 | ( | 25.0 | ) |  | 1.8 | 0.6194 |
|  | Autoimmune disease | |  | 20 | ( | 16.9 | ) |  | 9 | ( | 17.3 | ) |  | 1.4 | 0.4332 |  | 1 | ( | 12.5 | ) |  | 0.7 | > 0.9999 |
|  | Hypertension | |  | 6 | ( | 5.1 | ) |  | 4 | ( | 7.7 | ) |  | 2.1 | 0.2747 |  | 1 | ( | 12.5 | ) |  | 2.7 | 0.3756 |
|  | Heart disease | |  | 8 | ( | 6.8 | ) |  | 3 | ( | 5.8 | ) |  | 1.1 | > 0.9999 |  | 0 | ( | 0.0 | ) |  | 0.0 | > 0.9999 |
|  | Dyslipidemia | |  | 5 | ( | 4.2 | ) |  | 4 | ( | 7.7 | ) |  | 2.5 | 0.2323 |  | 0 | ( | 0.0 | ) |  | 0.0 | > 0.9999 |
|  | Hematological disease | |  | 4 | ( | 3.4 | ) |  | 3 | ( | 5.8 | ) |  | 2.3 | 0.3706 |  | 0 | ( | 0.0 | ) |  | 0.0 | > 0.9999 |
|  |  | Bone marrow transplantation | | 2 | ( | 1.7 | ) |  | 1 | ( | 1.9 | ) |  | 1.5 | > 0.9999 |  | 0 | ( | 0.0 | ) |  | 0.0 | > 0.9999 |
|  | Kideny disease | |  | 2 | ( | 1.7 | ) |  | 0 | ( | 0.0 | ) |  | 0.0 | > 0.9999 |  | 2 | ( | 25.0 | ) |  | 19.5 | 0.0200 |
|  | Hyperuricemia | |  | 2 | ( | 1.7 | ) |  | 1 | ( | 1.9 | ) |  | 1.5 | > 0.9999 |  | 0 | ( | 0.0 | ) |  | 0.0 | > 0.9999 |
|  | Psychiatric diseases | |  | 2 | ( | 1.7 | ) |  | 1 | ( | 1.9 | ) |  | 1.5 | > 0.9999 |  | 1 | ( | 12.5 | ) |  | 8.4 | 0.1786 |
|  | Neurological diseases | |  | 3 | ( | 2.5 | ) |  | 1 | ( | 1.9 | ) |  | 1.0 | > 0.9999 |  | 0 | ( | 0.0 | ) |  | 0.0 | > 0.9999 |
|  | Peripheral vascular disease | | | 1 | ( | 0.8 | ) |  | 0 | ( | 0.0 | ) |  | 0.0 | > 0.9999 |  | 0 | ( | 0.0 | ) |  | 0.0 | > 0.9999 |
|  | Endocine disease | |  | 3 | ( | 2.5 | ) |  | 1 | ( | 1.9 | ) |  | 1.0 | > 0.9999 |  | 0 | ( | 0.0 | ) |  | 0.0 | > 0.9999 |
|  | Cancer except the digestive or hematologic system | | | 7 | ( | 5.9 | ) |  | 0 | ( | 0.0 | ) |  | 0.0 | 0.1927 |  | 1 | ( | 12.5 | ) |  | 2.3 | 0.4175 |
| Segments involved | |  |  |  |  |  |  |  |  |  |  |  |  |  |  |  |  |  |  |  |  |  |  |
|  | Large bowel only | |  | 84 | ( | 71.8 | ) |  | 32 | ( | 59.3 | ) |  | 1.7 | 0.3078 |  | 3 | ( | 37.5 | ) |  | 0.3 | 0.0500 |
|  |  | Right-sided colon only | | 62 | ( | 53.0 | ) |  | 22 | ( | 40.7 | ) |  | 1.1 | 0.8260 |  | 0 | ( | 0.0 | ) |  | 0.0 | 0.0062 |
|  |  | Left-sided colon only | | 14 | ( | 12.0 | ) |  | 8 | ( | 14.8 | ) |  | 1.9 | 0.2063 |  | 3 | ( | 37.5 | ) |  | 4.5 | 0.0764 |
|  |  | Rectum only |  | 1 | ( | 0.9 | ) |  | 0 | ( | 0.0 | ) |  | 0.0 | > 0.9999 |  | 0 | ( | 0.0 | ) |  | 0.0 | > 0.9999 |
|  |  | Righ- and left-sided colon | | 5 | ( | 4.3 | ) |  | 1 | ( | 1.9 | ) |  | 0.6 | > 0.9999 |  | 0 | ( | 0.0 | ) |  | 0.0 | > 0.9999 |
|  |  | Left-sided colon and rectum | | 1 | ( | 0.9 | ) |  | 0 | ( | 0.0 | ) |  | 0.0 | > 0.9999 |  | 0 | ( | 0.0 | ) |  | 0.0 | > 0.9999 |
|  |  | Throughout the large bowel | | 1 | ( | 0.9 | ) |  | 1 | ( | 1.9 | ) |  | 3.0 | > 0.9999 |  | 0 | ( | 0.0 | ) |  | 0.0 | > 0.9999 |
|  | Small bowel only | |  | 26 | ( | 22.2 | ) |  | 6 | ( | 11.1 | ) |  | 0.6 | 0.4410 |  | 1 | ( | 12.5 | ) |  | 0.5 | > 0.9999 |
|  |  | Ileum only |  | 12 | ( | 10.3 | ) |  | 2 | ( | 3.7 | ) |  | 0.5 | 0.5209 |  | 0 | ( | 0.0 | ) |  | 0.0 | > 0.9999 |
|  |  | Jejunum only |  | 8 | ( | 6.8 | ) |  | 2 | ( | 3.7 | ) |  | 0.7 | > 0.9999 |  | 1 | ( | 12.5 | ) |  | 2.0 | 0.4599 |
|  |  | Ileum and jejunum | | 6 | ( | 5.1 | ) |  | 2 | ( | 3.7 | ) |  | 1.0 | > 0.9999 |  | 0 | ( | 0.0 | ) |  | 0.0 | > 0.9999 |
|  | Combined | |  | 7 | ( | 6.0 | ) |  | 2 | ( | 3.7 | ) |  | 0.8 | > 0.9999 |  | 4 | ( | 50.0 | ) |  | 16.0 | 0.0020 |
|  |  | Ileum and right-sided colon | | 2 | ( | 1.7 | ) |  | 1 | ( | 1.9 | ) |  | 1.5 | > 0.9999 |  | 0 | ( | 0.0 | ) |  | 0.0 | > 0.9999 |
|  |  | Jejunum and right-sided colon | | 0 | ( | 0.0 | ) |  | 0 | ( | 0.0 | ) |  | n.d | > 0.9999 |  | 1 | ( | 12.5 | ) |  | n.d | 0.0640 |
|  |  | Ileum, right- and left-sided colon | | 2 | ( | 1.7 | ) |  | 0 | ( | 0.0 | ) |  | 0.0 | > 0.9999 |  | 1 | ( | 12.5 | ) |  | 8.4 | 0.1813 |
|  |  | Jejunum, ileum, right- and left-sided colon | | 1 | ( | 0.9 | ) |  | 1 | ( | 1.9 | ) |  | 3.0 | > 0.9999 |  | 1 | ( | 12.5 | ) |  | 16.9 | 0.1244 |
|  |  | Esophagus, stomach, small bowel | | 2 | ( | 1.7 | ) |  | 0 | ( | 0.0 | ) |  | 0.0 | > 0.9999 |  | 1 | ( | 12.5 | ) |  | 8.4 | 0.1813 |
|  |  | Esophagus, stomach, small bowel, and colon | | 0 | ( | 0.0 | ) |  | 0 | ( | 0.0 | ) |  | n.d | > 0.9999 |  | 1 | ( | 12.5 | ) |  | n.d | 0.0640 |
| Complicating pnuematosis intestinalis | | |  | 20 | ( | 16.8 | ) |  | 2 | ( | 3.7 | ) |  | 0.3 | 0.0679 |  | 4 | ( | 50.0 | ) |  | 5.0 | 0.0412 |
| Death |  |  |  | 2 | ( | 1.7 | ) |  | 1 | ( | 1.9 | ) |  | 1.5 | > 0.9999 |  | 5 | ( | 62.5 | ) |  | 97.5 | < 0.0001 |
