# Supplementary material for: Multicenter epidemiological survey of pneumatosis intestinalis in Japan
Source: BMC Gastroenterol. 2022 May 31;22:272. doi: 10.1186/s12876-022-02343-5 (PMC9153137; doi:10.1186/s12876-022-02343-5)
Supplement: Supplementary file 6 — Additional file 6. Supplementary Table 6. [file 12876_2022_2343_MOESM6_ESM.docx]

| Supplementary Table 6. Prognosis of pneumatosis intestinalis | | | | | | | | | | | | | | | | | |
| --- | --- | --- | --- | --- | --- | --- | --- | --- | --- | --- | --- | --- | --- | --- | --- | --- | --- |
|  |  |  |  | Survival | | | | |  | Death | | | | |  | Crude  OR | *P* |
|  |  |  |  | n | | ( | % | ) |  | n | | ( | % | ) |  |  |  |
| Characteristics | |  |  | Presence | Absence |  |  |  |  | Presence | Absence |  |  |  |  |  |  |
| No.patients |  |  |  | 159 | | ( | 95.2 | ) |  | 8 | | ( | 4.8 | ) |  |  |  |
| Men/women |  |  |  | 80/79 | | ( | 1.0 | ) |  | 7/1 | | ( | 7.0 | ) |  | 6.9 | 0.0657 |
| Median age of onset (y) | |  |  | 65 (range 9-87) | | | | |  | 64 (range 26-91) | |  |  |  |  |  | 0.5813 |
| Exposure to organic solvents | | |  | 2 | 157 | ( | 1.3 | ) |  | 0 | 8 | ( | 0.0 | ) |  | 0.0 | > 0.9999 |
| Medications used | | |  | 116 | 30 | ( | 79.5 | ) |  | 6 | 0 | ( | 100.0 | ) |  | n.d | 0.5988 |
|  | Corticosteroid |  |  | 44 | 102 | ( | 30.1 | ) |  | 1 | 5 | ( | 16.7 | ) |  | 0.5 | 0.6704 |
|  | Antidiabetics |  |  | 28 | 118 | ( | 19.2 | ) |  | 1 | 5 | ( | 16.7 | ) |  | 0.8 | > 0.9999 |
|  |  | α-glucosidase inhibitors | | 22 | 124 | ( | 15.1 | ) |  | 1 | 5 | ( | 16.7 | ) |  | 1.1 | > 0.9999 |
|  |  | Sulfonylurea |  | 6 | 140 | ( | 4.1 | ) |  | 0 | 6 | ( | 0.0 | ) |  | 0.0 | > 0.9999 |
|  |  | Glinide |  | 3 | 143 | ( | 2.1 | ) |  | 0 | 6 | ( | 0.0 | ) |  | 0.0 | > 0.9999 |
|  |  | Insulin |  | 3 | 143 | ( | 2.1 | ) |  | 0 | 6 | ( | 0.0 | ) |  | 0.0 | > 0.9999 |
|  |  | Biguanide |  | 1 | 145 | ( | 0.7 | ) |  | 0 | 6 | ( | 0.0 | ) |  | 0.0 | > 0.9999 |
|  |  | Dipeptidyl peptidase 4 inhibitor | | 1 | 145 | ( | 0.7 | ) |  | 0 | 6 | ( | 0.0 | ) |  | 0.0 | > 0.9999 |
|  | Immunosuppressants | |  | 14 | 132 | ( | 9.6 | ) |  | 2 | 4 | ( | 33.3 | ) |  | 4.7 | 0.1215 |
|  | Anti-cancer agents | |  | 8 | 138 | ( | 5.5 | ) |  | 1 | 5 | ( | 16.7 | ) |  | 3.5 | 0.3110 |
|  | Antihypertensives | |  | 17 | 129 | ( | 11.6 | ) |  | 2 | 4 | ( | 33.3 | ) |  | 3.8 | 0.1637 |
|  |  | Calcium antagonist | | 10 | 136 | ( | 6.8 | ) |  | 0 | 6 | ( | 0.0 | ) |  | 0.0 | > 0.9999 |
|  |  | β-blocker |  | 8 | 138 | ( | 5.5 | ) |  | 0 | 6 | ( | 0.0 | ) |  | 0.0 | > 0.9999 |
|  |  | Angiotensin II receptor blocker | | 8 | 138 | ( | 5.5 | ) |  | 1 | 5 | ( | 16.7 | ) |  | 3.5 | 0.3110 |
|  |  | Angiotensin converting enzyme inhibitor | | 1 | 145 | ( | 0.7 | ) |  | 0 | 6 | ( | 0.0 | ) |  | 0.0 | > 0.9999 |
|  |  | α-blocker |  | 0 | 146 | ( | 0.0 | ) |  | 1 | 5 | ( | 16.7 | ) |  | n.d | 0.0395 |
|  | Diuretics |  |  | 4 | 142 | ( | 2.7 | ) |  | 1 | 5 | ( | 16.7 | ) |  | 7.1 | 0.1846 |
|  | Digitalis |  |  | 4 | 142 | ( | 2.7 | ) |  | 0 | 6 | ( | 0.0 | ) |  | 0.0 | > 0.9999 |
|  | Antiarrythmics |  |  | 1 | 145 | ( | 0.7 | ) |  | 1 | 5 | ( | 16.7 | ) |  | 29.0 | 0.0776 |
|  | Vasodilators |  |  | 3 | 143 | ( | 2.1 | ) |  | 1 | 5 | ( | 16.7 | ) |  | 9.5 | 0.1502 |
|  | Antithrombotics | |  | 12 | 134 | ( | 8.2 | ) |  | 0 | 6 | ( | 0.0 | ) |  | 0.0 | > 0.9999 |
|  |  | Anticoagulants | | 4 | 142 | ( | 2.7 | ) |  | 0 | 6 | ( | 0.0 | ) |  | 0.0 | > 0.9999 |
|  |  | Antiplatelets |  | 9 | 137 | ( | 6.2 | ) |  | 0 | 6 | ( | 0.0 | ) |  | 0.0 | > 0.9999 |
|  | Bronchodilators | |  | 3 | 143 | ( | 2.1 | ) |  | 0 | 6 | ( | 0.0 | ) |  | 0.0 | > 0.9999 |
|  | Gastric acid secretion inhibitors | | | 19 | 127 | ( | 13.0 | ) |  | 1 | 5 | ( | 16.7 | ) |  | 1.3 | 0.5776 |
|  |  | Proton pump inhibitors | | 14 | 132 | ( | 9.6 | ) |  | 1 | 5 | ( | 16.7 | ) |  | 1.9 | 0.4698 |
|  |  | Histamine-2 receptor antagonists | | 6 | 140 | ( | 4.1 | ) |  | 0 | 6 | ( | 0.0 | ) |  | 0.0 | > 0.9999 |
|  | 5-aminosalicylates or salicylazosulfapyridine | | | 15 | 131 | ( | 10.3 | ) |  | 0 | 6 | ( | 0.0 | ) |  | 0.0 | > 0.9999 |
|  | Biologics |  |  | 0 | 146 | ( | 0.0 | ) |  | 0 | 6 | ( | 0.0 | ) |  | n.d | > 0.9999 |
|  | Nonsteroidal antiinflammatory drugs | | | 3 | 143 | ( | 2.1 | ) |  | 0 | 6 | ( | 0.0 | ) |  | 0.0 | > 0.9999 |
|  | Antibiotics |  |  | 8 | 138 | ( | 5.5 | ) |  | 1 | 5 | ( | 16.7 | ) |  | 3.5 | 0.3110 |
|  |  | Trimethoprim-sulfamethoxazole | | 5 | 141 | ( | 3.4 | ) |  | 1 | 5 | ( | 16.7 | ) |  | 5.6 | 0.2179 |
|  | Laxatives |  |  | 7 | 139 | ( | 4.8 | ) |  | 3 | 3 | ( | 50.0 | ) |  | 19.9 | 0.0038 |
|  | Bisphophonates | |  | 2 | 144 | ( | 1.4 | ) |  | 0 | 6 | ( | 0.0 | ) |  | 0.0 | > 0.9999 |
|  | Statins / ezetimib/ fibrates | |  | 14 | 132 | ( | 9.6 | ) |  | 0 | 6 | ( | 0.0 | ) |  | 0.0 | > 0.9999 |
|  | Hypnotics |  |  | 2 | 144 | ( | 1.4 | ) |  | 0 | 6 | ( | 0.0 | ) |  | 0.0 | > 0.9999 |
|  | Psychotropics | |  | 5 | 141 | ( | 3.4 | ) |  | 1 | 5 | ( | 16.7 | ) |  | 5.6 | 0.2179 |
|  | Prostatic hypertrophy drugs | |  | 3 | 143 | ( | 2.1 | ) |  | 1 | 5 | ( | 16.7 | ) |  | 9.5 | 0.1502 |
|  | Allopurinol / benzbromaron | |  | 3 | 143 | ( | 2.1 | ) |  | 0 | 6 | ( | 0.0 | ) |  | 0.0 | > 0.9999 |
|  | Levothyroxine | |  | 4 | 142 | ( | 2.7 | ) |  | 0 | 6 | ( | 0.0 | ) |  | 0.0 | > 0.9999 |
|  | Herbal medicine | |  | 6 | 140 | ( | 4.1 | ) |  | 1 | 5 | ( | 16.7 | ) |  | 4.7 | 0.2501 |
| Comobidities and/or past medical history | | | | 132 | 26 | ( | 83.5 | ) |  | 7 | 1 | ( | 87.5 | ) |  | 1.4 | > 0.9999 |
|  | Gastroduodenal diseases | |  | 44 | 114 | ( | 27.8 | ) |  | 0 | 8 | ( | 0.0 | ) |  | 0.0 | 0.1112 |
|  |  | Inflammatory bowel disease | | 17 | 141 | ( | 10.8 | ) |  | 0 | 8 | ( | 0.0 | ) |  | 0.0 | > 0.9999 |
|  |  |  | Ulcerative colitis | 13 | 145 | ( | 8.2 | ) |  | 0 | 8 | ( | 0.0 | ) |  | 0.0 | > 0.9999 |
|  |  |  | Crohn's disease | 1 | 157 | ( | 0.6 | ) |  | 0 | 8 | ( | 0.0 | ) |  | 0.0 | > 0.9999 |
|  |  |  | Behcet's disease | 1 | 157 | ( | 0.6 | ) |  | 0 | 8 | ( | 0.0 | ) |  | 0.0 | > 0.9999 |
|  |  | Carcinoma |  | 13 | 145 | ( | 8.2 | ) |  | 0 | 8 | ( | 0.0 | ) |  | 0.0 | > 0.9999 |
|  |  |  | Esophegeal carcinoma | 1 | 157 | ( | 0.6 | ) |  | 0 | 8 | ( | 0.0 | ) |  | 0.0 | > 0.9999 |
|  |  |  | Gastric carcinoma | 3 | 155 | ( | 1.9 | ) |  | 0 | 8 | ( | 0.0 | ) |  | 0.0 | > 0.9999 |
|  |  |  | Colorectal carcinoma | 9 | 149 | ( | 5.7 | ) |  | 0 | 8 | ( | 0.0 | ) |  | 0.0 | > 0.9999 |
|  |  | Colorectal polyp | | 5 | 153 | ( | 3.2 | ) |  | 0 | 8 | ( | 0.0 | ) |  | 0.0 | > 0.9999 |
|  |  | Bowel obstruction | | 3 | 155 | ( | 1.9 | ) |  | 0 | 8 | ( | 0.0 | ) |  | 0.0 | > 0.9999 |
|  |  | Others |  | 5 | 153 | ( | 3.2 | ) |  | 0 | 8 | ( | 0.0 | ) |  | 0.0 | > 0.9999 |
|  |  |  | Esophegeal candidiasis | 1 | 157 | ( | 0.6 | ) |  | 0 | 8 | ( | 0.0 | ) |  | 0.0 | > 0.9999 |
|  |  |  | Gastroesophageal reflux disease | 1 | 157 | ( | 0.6 | ) |  | 0 | 8 | ( | 0.0 | ) |  | 0.0 | > 0.9999 |
|  |  |  | Peptic ulcer disease | 2 | 156 | ( | 1.3 | ) |  | 0 | 8 | ( | 0.0 | ) |  | 0.0 | > 0.9999 |
|  |  |  | Ischemic colitis | 1 | 157 | ( | 0.6 | ) |  | 0 | 8 | ( | 0.0 | ) |  | 0.0 | > 0.9999 |
|  | Hepatobiliarypancreatic disease | | | 9 | 149 | ( | 5.7 | ) |  | 1 | 7 | ( | 12.5 | ) |  | 2.4 | 0.3984 |
|  |  | Hepatic hemangioma | | 1 | 157 | ( | 0.6 | ) |  | 0 | 8 | ( | 0.0 | ) |  | 0.0 | > 0.9999 |
|  |  | Chronic hepatitis | | 1 | 157 | ( | 0.6 | ) |  | 0 | 8 | ( | 0.0 | ) |  | 0.0 | > 0.9999 |
|  |  | Cirrhosis |  | 0 | 158 | ( | 0.0 | ) |  | 1 | 7 | ( | 12.5 | ) |  | n.d | 0.0482 |
|  |  | Hepatic carcinoma | | 1 | 157 | ( | 0.6 | ) |  | 0 | 8 | ( | 0.0 | ) |  | 0.0 | > 0.9999 |
|  |  | Cholecystitis |  | 2 | 156 | ( | 1.3 | ) |  | 0 | 8 | ( | 0.0 | ) |  | 0.0 | > 0.9999 |
|  |  | Chroinc pancreatitis | | 1 | 157 | ( | 0.6 | ) |  | 0 | 8 | ( | 0.0 | ) |  | 0.0 | > 0.9999 |
|  | Diabetes mellitus | |  | 31 | 127 | ( | 19.6 | ) |  | 1 | 7 | ( | 12.5 | ) |  | 0.6 | > 0.9999 |
|  | Chronic lung disease | |  | 27 | 131 | ( | 17.1 | ) |  | 1 | 7 | ( | 12.5 | ) |  | 0.7 | > 0.9999 |
|  | Autoimmune disease | |  | 32 | 126 | ( | 20.3 | ) |  | 0 | 8 | ( | 0.0 | ) |  | 0.0 | 0.3559 |
|  | Hypertension |  |  | 11 | 147 | ( | 7.0 | ) |  | 2 | 6 | ( | 25.0 | ) |  | 4.5 | 0.1213 |
|  | Heart disease |  |  | 10 | 148 | ( | 6.3 | ) |  | 1 | 7 | ( | 12.5 | ) |  | 2.1 | 0.4293 |
|  | Dyslipidemia |  |  | 9 | 149 | ( | 5.7 | ) |  | 0 | 8 | ( | 0.0 | ) |  | 0.0 | > 0.9999 |
|  | Hematological disease | |  | 6 | 152 | ( | 3.8 | ) |  | 1 | 7 | ( | 12.5 | ) |  | 3.6 | 0.2970 |
|  |  | Bone marrow transplantation | | 2 | 156 | ( | 1.3 | ) |  | 1 | 7 | ( | 12.5 | ) |  | 11.1 | 0.1385 |
|  | Kideny disease | |  | 2 | 156 | ( | 1.3 | ) |  | 2 | 6 | ( | 25.0 | ) |  | 26.0 | 0.0117 |
|  | Hyperuricemia | |  | 3 | 155 | ( | 1.9 | ) |  | 0 | 8 | ( | 0.0 | ) |  | 0.0 | > 0.9999 |
|  | Psychiatric diseases | |  | 3 | 155 | ( | 1.9 | ) |  | 1 | 7 | ( | 12.5 | ) |  | 7.4 | 0.1808 |
|  | Neurological diseases | |  | 4 | 154 | ( | 2.5 | ) |  | 0 | 8 | ( | 0.0 | ) |  | 0.0 | > 0.9999 |
|  | Peripheral vascular disease | |  | 1 | 157 | ( | 0.6 | ) |  | 0 | 8 | ( | 0.0 | ) |  | 0.0 | > 0.9999 |
|  | Endocine disease | |  | 2 | 156 | ( | 1.3 | ) |  | 0 | 8 | ( | 0.0 | ) |  | 0.0 | > 0.9999 |
|  | Cancer except the digestive or hematologic system | | | 7 | 151 | ( | 4.4 | ) |  | 1 | 7 | ( | 12.5 | ) |  | 3.1 | 0.3323 |
| Segments involved | |  |  |  |  |  |  |  |  |  |  |  |  |  |  |  |  |
|  | Large bowel only | |  | 117 | 40 | ( | 74.5 | ) |  | 1 | 7 | ( | 12.5 | ) |  | 0.0 | 0.0007 |
|  |  | Right-sided colon only | | 83 | 74 | ( | 52.9 | ) |  | 0 | 8 | ( | 0.0 | ) |  | 0.0 | 0.0007 |
|  |  | Left-sided colon only | | 25 | 132 | ( | 15.9 | ) |  | 0 | 8 | ( | 0.0 | ) |  | 0.0 | 0.6085 |
|  |  | Rectum only |  | 1 | 156 | ( | 0.6 | ) |  | 0 | 8 | ( | 0.0 | ) |  | 0.0 | > 0.9999 |
|  |  | Righ- and left-sided colon | | 6 | 151 | ( | 3.8 | ) |  | 0 | 8 | ( | 0.0 | ) |  | 0.0 | > 0.9999 |
|  |  | Left-sided colon and rectum | | 1 | 156 | ( | 0.6 | ) |  | 0 | 8 | ( | 0.0 | ) |  | 0.0 | > 0.9999 |
|  |  | Throughout the large bowel | | 1 | 156 | ( | 0.6 | ) |  | 1 | 7 | ( | 12.5 | ) |  | 22.3 | 0.0949 |
|  | Small bowel only | |  | 32 | 125 | ( | 20.4 | ) |  | 1 | 7 | ( | 12.5 | ) |  | 0.6 | > 0.9999 |
|  |  | Ileum only |  | 14 | 143 | ( | 8.9 | ) |  | 0 | 8 | ( | 0.0 | ) |  | 0.0 | > 0.9999 |
|  |  | Jejunum only |  | 10 | 147 | ( | 6.4 | ) |  | 1 | 7 | ( | 12.5 | ) |  | 2.1 | 0.4313 |
|  |  | Ileum and jejunum | | 8 | 149 | ( | 5.1 | ) |  | 0 | 8 | ( | 0.0 | ) |  | 0.0 | > 0.9999 |
|  | Combined |  |  | 7 | 150 | ( | 4.5 | ) |  | 6 | 2 | ( | 75.0 | ) |  | 64.3 | < 0.0001 |
|  |  | Ileum and right-sided colon | | 3 | 154 | ( | 1.9 | ) |  | 0 | 8 | ( | 0.0 | ) |  | 0.0 | > 0.9999 |
|  |  | Jejunum and right-sided colon | | 0 | 157 | ( | 0.0 | ) |  | 1 | 7 | ( | 12.5 | ) |  | n.d | 0.0485 |
|  |  | Ileum, right- and left-sided colon | | 1 | 156 | ( | 0.6 | ) |  | 2 | 6 | ( | 25.0 | ) |  | 52.0 | 0.0061 |
|  |  | Jejunum, ileum, right- and left-sided colon | | 1 | 156 | ( | 0.6 | ) |  | 2 | 6 | ( | 25.0 | ) |  | 52.0 | 0.0061 |
|  |  | Esophagus, stomach, small bowel | | 2 | 155 | ( | 1.3 | ) |  | 0 | 8 | ( | 0.0 | ) |  | 0.0 | > 0.9999 |
|  |  | Esophagus, stomach, small bowel, and colon | | 0 | 157 | ( | 0.0 | ) |  | 1 | 7 | ( | 12.5 | ) |  | n.d | 0.0485 |
| Complicating pnuematosis intestinalis | | |  | 20 | 97 | ( | 17.1 | ) |  | 6 | 2 | ( | 75.0 | ) |  | 14.6 | 0.0010 |
| Treatment |  |  |  |  |  |  |  |  |  |  |  |  |  |  |  |  |  |
|  | Medical treatment or observation | | | 111 | 48 | ( | 69.8 | ) |  | 6 | 2 | ( | 75.0 | ) |  | 1.3 | > 0.9999 |
|  | Oxygene therapy | |  | 34 | 125 | ( | 21.4 | ) |  | 1 | 7 | ( | 12.5 | ) |  | 0.5 | > 0.9999 |
|  |  | Hyperbaric |  | 16 | 143 | ( | 10.1 | ) |  | 0 | 8 | ( | 0.0 | ) |  | 0.0 | > 0.9999 |
|  |  | Conventional |  | 18 | 141 | ( | 11.3 | ) |  | 1 | 7 | ( | 12.5 | ) |  | 1.1 | > 0.9999 |
|  | Endoscopic therapy | |  | 3 | 156 | ( | 1.9 | ) |  | 0 | 8 | ( | 0.0 | ) |  | 0.0 | > 0.9999 |
|  | Surgery |  |  | 11 | 148 | ( | 6.9 | ) |  | 1 | 7 | ( | 12.5 | ) |  | 1.9 | 0.4566 |
